# Bounds on Hankel Determinants with Fekete-Szegö Parameter for Bazilević Functions

**DOI:** 10.12688/f1000research.173313.1

**Published:** 2026-02-14

**Authors:** Nathir Khaled, Abdul Rahman S.Juma

**Affiliations:** 1 Mathematics, University of Anbar, Ramadi, Al Anbar Governorate, Iraq

**Keywords:** Hankel determinant, Bazileviˇc function, subordination, Yamaguchi function, Ma-Minda function, Fekete-Szegö estimate

## Abstract

This paper presents a systematic investigation of a newly defined comprehensive subclass of analytic and univalent functions in the open unit disk. The introduced function class constitutes a broad generalization that incorporates several significant families, particularly Yamaguchi functions and starlike functions, which themselves exhibit fundamental properties within the established Bazilevič function hierarchy. The formal construction of this class employs the Ma-Minda framework through the application of subordination principles, set-theoretic operations, and analytical formulations involving infinite series and product combinations of specific geometric expressions.

The principal contributions of this work include the derivation of sharp coefficient bounds, complete solutions for the Fekete-Szegö problem with parameters, and precise estimates for Hankel determinants containing a real parameter. The proposed class demonstrates remarkable generality, as it systematically reduces to numerous well-established function classes when its parameters are specialized to particular values within their defined domains, thereby providing a unified approach to studying these classical families.

## 1. Introduction

Geometric Function Theory (GFT), a captivating branch of complex analysis, is dedicated to studying the geometric properties of analytic functions. Its importance is also observed across numerous mathematical and physical disciplines, such as ( q− ) calculus, special functions, orthogonal polynomials, and the theory of conformal mappings.

Let A be the family of analytic functions on the open unit disk Ω={z∈ℂ:|z|<1} , and let S be its subclass of normalized univalent functions of the form:

$$f\left(z\right)=z+\sum_{j=2}^{\infty }a_{j}z^{j},$$ (1)

satisfying the conditions f(0)=0 and $f^{\prime }\left(0\right)=1$ .

A cornerstone of GFT is the coefficient problem, which seeks to determine the possible values of the coefficients $a_{j}$ and the bounds of functionals constructed from them. This problem was famously initiated by Bieberbach’s 1916 conjecture that | $a_{j}$ | ≤j for all j≥2 . The eventual proof of this conjecture by de Branges in 1985 ^1^ underscored the profound depth of this area of research. The pursuit of sharp bounds for other coefficient functionals, such as the Fekete-Szegö functional and Hankel determinants, remains an active and central theme in GFT.

Prominent subclasses of S , including starlike, convex, close-to-convex, and Yamaguchi functions, are defined based on the geometric characteristics of their image domains. The class of Bazilević functions, introduced in, ^2^ represents one of the largest known subclasses of S .

A powerful tool in defining these classes is the principle of subordination (denoted f≺F ). For two functions ( f,F∈A ), we say f is subordinate to F if there exists an analytic function:

$$s\left(z\right)=s_{1}z+s_{2}z^{2}+s_{3}z^{3}\ldots$$ (2)

with s(0)=0 and |s(z)|<1 such that f(z)=F(s(z)). If F is univalent, this is equivalent to f(0)=F(0) and f ( Ω ) ⊂ F(Ω) .

The Carathéodory class, consisting of functions $p\left(z\right)=1+p_{1}z+p_{2}z^{2}+\ldots$ with a positive real part in Ω , plays a pivotal role in solving many problems in GFT. The Möbius function $m_{0}\left(z\right)=\left(1+z\right)/\left(1-z\right)$ serves as the extremal function for this class.

In a significant unification effort, Ma and Minda ^3^ introduced a general class using a function b(z) that is analytic, univalent, has a positive real part, and is characterized by a Taylor series $b\left(z\right)=1+B_{1}z+B_{2}z^{2}+\ldots$ with $B_{1}$ > 0. This framework elegantly encapsulates many previously studied classes.

In this paper, we build upon these foundations to define a new and comprehensive subclass Λ(δ , b) of analytic-univalent functions using a combination of concepts from Bazilević, Yamaguchi, and starlike functions, subordinated to a Ma-Minda function. We then proceed to derive sharp coefficient bounds, Fekete-Szegö inequalities, and Hankel determinant estimates for functions belonging to this class, demonstrating that it generalizes several important families of functions previously studied in the literature.

### 1.1 Relevant subsets of analytic functions

A function Ҩ is called a Carathéodory function if it is analytic in the open unit disk Ω , has a positive real part, and is normalized such that Ҩ(0)=1 . The class of all functions is symbolized by P . Formally, a function Ҩ belongs to P if it can be expressed as:

$$Ҩ\left(z\right)=1+\sum_{j=1}^{\infty }p_{j}z^{j},Re(Ҩ\left(z)\right)>0\;\text{for}\;\mathrm{all}\;z\;\in \;\Omega .$$ (3)

Over the decades, this class has proven to be fundamental in solving numerous problems in GFT. A canonical and extremal function within the class P is the Möbius function:

$$m_{0}\left(z\right)=\frac{1+z}{1-z}=1+2\sum_{j=1}^{\infty }z^{j}\;\left(z\;\in \;\Omega \right),$$ (4)

there exists a fundamental relationship between Carathéodory functions Ҩ(z) and functions s(z) analytic in Ω with |s(z)|<1 and s(0)=0 . This relationship is given by the following transformation:

$$Ҩ\left(z\right)=\frac{1+s\left(z\right)}{1-s\left(z\right)}⟺s\left(z\right)=\frac{Ҩ\left(z\right)-1}{Ҩ\left(z\right)+1}\;\left(z\;\in \;\Omega \right),$$ (5)

substituting the Taylor series for $p\left(z\right)=1+p_{1}z+p_{2}z^{2}+\ldots$ into this relation yields the series expansion for s(z) :

$$s\left(z\right)=\frac{1}{2}\left[p_{1}z+\left(p_{2}-\frac{p_{1}^{2}}{2}\right)z^{2}+\left(p_{3}-p_{1}p_{2}+\frac{p_{1}^{3}}{4}\right)z^{3}+\ldots \right].$$ (6)

A significant unification of several subclasses of starlike and convex functions was achieved by Ma and Minda ^3^ in 1994. They introduced a function b(z) , which is analytic and univalent with a series expansion of the form:

$$b\left(z\right)=1+\beta_{1}z+\beta_{2}z^{2}+\beta_{3}z^{3}+\ldots \left(\beta_{1}>0,\beta_{k}\;\in \;\mathbb{R},z\;\in \;\Omega \right).$$ (7)

This function b(z) has the following key properties: ( Re(b(z)))>0,b(0)=1 , $b^{\prime }\left(0\right)>0$ , b(z) maps the unit disk Ω onto a domain that is starlike with respect to 1 and symmetric about the real axis. By composing this function with s(z) , we obtain:

$$\begin{array}{c} b\left(s\left(z\right)\right)=1+\beta_{1}s\left(z\right)+\beta_{2}{\left(s\left(z\right)\right)}^{2}+\beta_{3}{\left(s\left(z\right)\right)}^{3}+\ldots \\ =1+\frac{\beta_{1}}{2}p_{1}z+\left[\frac{\beta_{1}}{2}\left(p_{2}-\frac{p_{1}^{2}}{2}\right)+\frac{\beta_{2}}{4}p_{1}^{2}\right]\;z^{2}\\ +\left[\frac{\beta_{1}}{2}\left(p_{3}-p_{1}p_{2}+\frac{p_{1}^{3}}{4}\right)+\frac{\beta_{2}}{2}p_{1}\left(p_{2}-\frac{p_{1}^{2}}{2}\right)+\frac{\beta_{3}}{8}p_{1}^{3}\right]z^{3}+\ldots \end{array}$$ (8)

If a function f∈S maps the unit disk Ω onto a starlike domain, then f is classified as a starlike function. This geometric property is characterized by the analytic condition:

$$f\;\in \;\mathrm{ST}=\left\{f:f\;\in \;S,Re\left(\frac{zf^{\prime }\left(z\right)}{f\left(z\right)}\right)>0,\text{and}\;z\;\in \;\Omega \;\right\}.$$ (9)

The extremal function for the class ST is the Koebe function:

$$k\left(z\right)=\frac{z}{{\left(1-z\right)}^{2}}.$$

The class ST was first presented via Alexander, ^4^ though its corresponding geometric property was actually characterized earlier via Nevanlinna in 1921. ^5^ Research into starlike functions has since expanded significantly, leading to diverse formulations and applications, as explored by authors such as Lasode and Opoola. ^6^ Later, in 1956, Yamaguchi ^7^ defined a specific subclass of S characterized by the following condition:

$$f\;\in \;\Upsilon =\left\{f:f\;\in \;S,Re\left(f^{\prime }\left(z\right)\right)>0,\text{and}\;z\;\in \;\Omega \;\right\}.$$ (10)

Various properties of functions in the Yamaguchi class Υ -such as univalence, radii problems, partial sums, and growth, distortion, and inclusion theorems-have been demonstrated in the literature on GFT. ^7– 9^ Another significant subclass is B(δ,γ,Ҩ,h) , introduced by Bazilevič. ^2^ Recognized as one of the largest subclasses of S , its functions are specified by the integral representation:

$$f\left(z\right)={\left\{\left(\delta +\mathrm{i\gamma}\right)\int_{0}^{z}Ҩ\left(t\right)h{\left(t\right)}^{\gamma }t^{-\left(1-\mathrm{i\gamma}\right)}\mathrm{dt}\right\}}^{\frac{1}{\delta +\mathrm{i\gamma}}},$$

where: δ >0 , γ∈ℝ , h∈ST (a starlike function), Ҩ∈P (a Carathéodory function), and we employ the principal value for all powers. Singh ^10^ introduced two important subclasses:

$$B\left(\delta ,0,Ҩ,h\right)=B\left(0\right),B\left(\delta ,0,Ҩ,z\right)=B_{1}\left(0\right).$$

For: δ >0 and z∈Ω , these classes are characterized by the following conditions, respectively:

$$Re\frac{zf^{\prime }\left(z\right)f{\left(z\right)}^{\delta -1}}{h{\left(z\right)}^{\delta }}>0\;\text{and}\;Re\frac{zf^{\prime }\left(z\right)f{\left(z\right)}^{\delta -1}}{z^{\delta }}>0.$$ (11)

Furthermore, the following inclusion relationships hold:

$$B_{1}\left(\delta \right)\;\subset \;B\left(\delta \right)\;\subset \;B\left(\delta ,\gamma ,Ҩ,h\right),B_{1}\left(0\right)=B\left(0\right)=\mathrm{ST}.$$

Special cases for δ=1 are particularly noteworthy:

B(1) , characterized by $Re\left(\frac{zf^{\prime }\left(z\right)}{h\left(z\right)}\right)>0$ , represents the class of close-to-convex functions.

$B_{1}\left(1\right)$ , characterized by $Re\left(f^{\prime }\left(z\right)\right)>0$ , represents the class of bounded turning functions.

## 2. Lemmas

Lemma 3.1. ^5^Let Ҩ∈CR. Then

$$\left|p_{j}\right|\;≦\;2\forall j\;\in \;\left\{1,2,3,4,\ldots \right\}$$

The function (4) has a sharp inequality.

Lemma 3.2. ^11^Let Ҩ∈CR. Then

$$\left|p_{2}-\lambda \frac{p_{1}^{2}}{2}\right|\;≦\;\left\{ \begin{array}{ccc} 2\left(1-\lambda \right) & \text{when} & \lambda \;≦\;0\\ 2 & \text{when} & 0\;≦\;\lambda \;≦\;2\\ 2\left(\lambda -1\right) & \text{when} & \lambda \;≦\;2\\ 2\max\left\{1,|1-\lambda |\right\} & \text{when} & \lambda \;\in \;\mathbb{C}\; \end{array}\right.$$

Lemma 3.3. ^12^Let Ҩ∈CR. Then

$$\left|up_{1}^{3}-vp_{1}p_{2}+wp_{3}\right|\;≦\;2\left|u\right|+2\left|v-2u\right|+2\left|u-v+w\right|.$$

Lemma 3.4. ^13^Let Ҩ∈CR. Then for i,j∈{1,2,3,…},

$$\left|p_{i+j}-\mu p_{i}p_{j}\right|\;≦\;\left\{ \begin{array}{ccc} 2 & \text{when} & 0\;≦\;\mu \;≦\;1\\ 2\left|2\mu -1\right| & & \text{elsewhere} \end{array}\right..$$

## 3. Main results

### 3.1 A new class of analytic-univalent functions

A novel category of analytic univalent functions is introduced in this work. This class is developed by extending the foundational principles of Bazilevič functions and other established families, incorporating the theory of subordination.

Definition 1. The class Λ(δ,b) consists of all functions f∈A satisfying

$${\left(f^{\prime }\left(z\right)\right)}^{\delta }{\left(\frac{zf^{\prime }\left(z\right)}{f\left(z\right)}\right)}^{1-\delta }≺\;b\left(z\right),$$ (12)

with 0≤δ≤1 and where b(z) is the Ma-Minda function defined in (7).

Remark 1. This newly defined class provides a generalization of several established families. Its generality is demonstrated by the following examples:

1. When δ=1 and $b\left(z\right)=m_{0}\left(z\right)$ (see (4)), the subordination (12) simplifies to

$$f^{\prime }\left(z\right)≺\;m_{0}\left(z\right).$$

2. Yamaguchi Functions: Choosing δ=1 and $b\left(z\right)=m_{0}\left(z\right)$ from (4) yields the condition

$$f^{\prime }\left(z\right)≺\;m_{0}\left(z\right).$$

This is the defining property for the class of Yamaguchi functions, referenced in (10).

3. Starlike Functions: Taking δ=0 with $b\left(z\right)=m_{0}\left(z\right)$ from (4) leads to $\frac{zf^{\prime }\left(z\right)}{f\left(z\right)}≺\;m_{0}\left(z\right)$ .
  This subordination characterizes the family of starlike functions given in (9).

This study focuses on deriving sharp estimates for various problems within these classes. We establish upper bounds for the initial coefficients, the Fekete-Szegö functional, and specific Hankel determinants that incorporate τ>0 .

### 3.2 Coefficient estimates for set Λ(δ,b)

Theorem 1. If a function f∈A belongs to the class Λ(δ,b) , then

$$\left|a_{2}\right|\;\leq \;\frac{\beta_{1}}{1+\delta }$$ (13)

$$\left|a_{3}\right|\;\leq \;\frac{2\beta_{1}+\left|\beta_{2}\right|}{2+\delta }+\frac{\left(1-\delta \right)\beta_{1}^{2}}{{\left(1+\delta \right)}^{2}}$$ (14)

$$\left|a_{4}\right|\;\leq \;\frac{\beta_{1}+2\left|\beta_{2}\right|+\left|\beta_{3}\right|}{3+\delta }+\frac{\left(1-\delta \right)\left(2-\delta \right)\beta_{1}^{3}}{6{\left(1+\delta \right)}^{3}}+\frac{\left(1-\delta \right)\beta_{1}^{2}}{\left(1+\delta \right)\left(2+\delta \right)}+\frac{\left(\left(1-\delta \right)\beta_{1}\right)\left(\left|\beta_{2}\right|+\beta_{1}\right)}{\left(1+\delta \right)\left(2+\delta \right)}+\frac{{\left(1-\delta \right)}^{2}\beta_{1}^{3}}{2{\left(1+\delta \right)}^{3}}.$$ (15)

And

$$\begin{array}{c} \left|a_{5}\right|\;\leq \;\frac{5\beta_{1}+3\left|\beta_{2}\right|+3\left|\beta_{3}\right|+\left|\beta_{4}\right|}{\left(4+\delta \right)}+\frac{\left(1-\delta \right)\left(2-\delta \right)}{2}\left(\frac{\beta_{1}^{2}}{{\left(1+\delta \right)}^{2}}\right)\left(\frac{2\beta_{1}+\left|\beta_{2}\right|}{\left(2+\delta \right)}+\frac{\left(1-\delta \right)\beta_{1}^{2}}{2{\left(1+\delta \right)}^{2}}\right)+\frac{\left(1-\delta \right)\left(2-\delta \right)\left(3-\delta \right)\beta_{1}^{4}}{24{\left(1+\delta \right)}^{4}}\\ +\frac{\left(1-\delta \right)\beta_{1}}{\left(1+\delta \right)}\left(\frac{\beta_{1}+2\left|\beta_{2}\right|+\left|\beta_{3}\right|}{3+\delta }+\frac{\left(1-\delta \right)\left(2-\delta \right)\beta_{1}^{3}}{6{\left(1+\delta \right)}^{3}}+\frac{\left(1-\delta \right)\beta_{1}^{2}}{\left(1+\delta \right)\left(2+\delta \right)}+\frac{\left(\left(1-\delta \right)\beta_{1}\right)\left(\left|\beta_{2}\right|+\beta_{1}\right)}{\left(1+\delta \right)\left(2+\delta \right)}+\frac{{\left(1-\delta \right)}^{2}\beta_{1}^{3}}{2{\left(1+\delta \right)}^{3}}\right)\\ +\frac{\left(1-\delta \right)}{2}{\left(\frac{2\beta_{1}+\left|\beta_{2}\right|}{2+\delta }+\frac{\left(1-\delta \right)\beta_{1}^{2}}{{\left(1+\delta \right)}^{2}}\right)}^{2}. \end{array}$$ (16)

Proof. Let f∈A be a member of Λ(δ,b) . By the definition of subordination, there exists a Schwarz function such

$$\left(f^{\prime }\left(z\right)\right){\left(\frac{zf^{\prime }\left(z\right)}{f\left(z\right)}\right)}^{1-\delta }=b\left(s\left(z\right)\right)\;\left(z\;\in \;\Omega \right),$$ (17)

using the binomial theorem, the left-hand side of (17) can be expressed as the series:

$$\begin{array}{c} {\left(f^{\prime }\left(z\right)\right)}^{\delta }{\left(\frac{zf^{\prime }\left(z\right)}{f\left(z\right)}\right)}^{1-\delta }=1+\left(1+\delta \right)a_{2}z+\frac{1}{2}\left(2+\delta \right)\left[2a_{3}-\left(1-\delta \right)a_{2}^{2}\right]z^{2}\\ +\frac{\left(3+\delta \right)}{6}\left[\left(1-\delta \right)\left(2-\delta \right)a_{2}^{3}-6\left(1-\delta \right)a_{2}a_{3}+6a_{4}\right]z^{3}+\ldots , \end{array}$$ (18)

consequently, a comparison of the coefficients in (18) and (8) gives

$$a_{2}=\frac{\beta_{1}p_{1}}{2\left(1+\delta \right)},$$ (19)

$$a_{3}=\frac{\beta_{1}p_{2}}{2\left(2+\delta \right)}+\left[\frac{\beta_{2}-\beta_{1}}{4\left(2+\delta \right)}+\frac{\left(1-\delta \right)\beta_{1}^{2}}{8{\left(1+\delta \right)}^{2}}\right]p_{1}^{2},$$ (20)

$$\begin{array}{c} a_{4}=\frac{1}{2\left(3+\delta \right)}\left[\beta_{1}\left(p_{3}-p_{1}p_{2}+\frac{p_{1}^{3}}{4}\right)+\beta_{2}p_{1}\left(p_{2}-\frac{p_{1}^{2}}{2}\right)+\frac{\beta_{3}}{4}p_{1}^{3}\right]-\frac{\left(1-\delta \right)\left(2-\delta \right)\beta_{1}^{3}p_{1}^{3}}{48{\left(1+\delta \right)}^{3}}\\ +\frac{\left(1-\delta \right)\beta_{1}p_{1}}{2\left(1+\delta \right)}\left[\frac{\beta_{1}p_{2}}{2\left(2+\delta \right)}+\left(\frac{\beta_{2}-\beta_{1}}{4\left(2+\delta \right)}+\frac{\left(1-\delta \right)\beta_{1}^{2}}{8{\left(1+\delta \right)}^{2}}\right)p_{1}^{2}\right]. \end{array}$$ (21)

And

$$a_{5}=\frac{\beta_{1}}{2\left(4+\delta \right)}\left(p_{4}-p_{1}p_{3}-\frac{p_{2}^{2}}{2}+\frac{3p_{1}^{2}p_{2}}{4}-\frac{p_{1}^{4}}{8}\right)+\frac{\beta_{2}p_{1}}{2\left(4+\delta \right)}\left(p_{3}-p_{1}p_{2}+\frac{p_{1}^{3}}{4}\right)+\frac{\beta_{2}}{4\left(4+\delta \right)}{\left(p_{2}-\frac{p_{1}^{2}}{2}\right)}^{2}+\frac{3\beta_{3}p_{1}^{2}}{8\left(4+\delta \right)}\left(p_{2}-\frac{p_{1}^{2}}{2}\right)+\frac{\beta_{4}p_{1}^{4}}{16\left(4+\delta \right)}-\frac{\left(1-\delta \right)\left(2-\delta \right)}{2}{\left(\frac{\beta_{1}p_{1}}{2\left(1+\delta \right)}\right)}^{2}\left(\frac{\beta_{1}p_{2}}{2\left(2+\delta \right)}+\left[\frac{\beta_{2}-\beta_{1}}{4\left(2+\delta \right)}+\frac{\left(1-\delta \right)\beta_{1}^{2}}{8{\left(1+\delta \right)}^{2}}\right]p_{1}^{2}\right)+\frac{\left(1-\delta \right)\left(2-\delta \right)\left(3-\delta \right)}{24}{\left(\frac{\beta_{1}p_{1}}{2\left(1+\delta \right)}\right)}^{4}+\left(1-\delta \right)\left(\frac{\beta_{1}p_{1}}{2\left(1+\delta \right)}\right)\left(\frac{1}{2\left(3+\delta \right)}\left[\beta_{1}\left(p_{3}-p_{1}p_{2}+\frac{p_{1}^{3}}{4}\right)+\beta_{2}p_{1}\left(p_{2}-\frac{p_{1}^{2}}{2}\right)+\frac{\beta_{3}}{4}p_{1}^{3}\right]-\frac{\left(1-\delta \right)\left(2-\delta \right)\beta_{1}^{3}p_{1}^{3}}{48{\left(1+\delta \right)}^{3}}+\frac{\left(1-\delta \right)\beta_{1}p_{1}}{2\left(1+\delta \right)}\left[\frac{\beta_{1}p_{2}}{2\left(2+\delta \right)}+\left(\frac{\beta_{2}-\beta_{1}}{4\left(2+\delta \right)}+\frac{\left(1-\delta \right)\beta_{1}^{2}}{8{\left(1+\delta \right)}^{2}}\right)p_{1}^{2}\right]\right)+\frac{\left(1-\delta \right)}{2}{\left(\frac{\beta_{1}p_{2}}{2\left(2+\delta \right)}+\left[\frac{\beta_{2}-\beta_{1}}{4\left(2+\delta \right)}+\frac{\left(1-\delta \right)\beta_{1}^{2}}{8{\left(1+\delta \right)}^{2}}\right]p_{1}^{2}\right)}^{2}.$$ (22)

Apparently, (19) gives

$$\left|a_{2}\right|=\frac{\beta_{1}}{2\left(1+\delta \right)}\left|p_{1}\right|.$$

Utilizing Lemma 1, we obtain the result given in (13). Additionally, from (20) it follows that

$$a_{3}\;\leq \;\frac{\beta_{1}\left|p_{2}\right|}{2\left(2+\delta \right)}+\left[\frac{\left|\beta_{2}\right|+\beta_{1}}{4\left(2+\delta \right)}+\frac{\left(1-\delta \right)\beta_{1}^{2}}{8{\left(1+\delta \right)}^{2}}\right]{\left|p_{1}\right|}^{2}.$$

The result in (14) follows from an application of Lemma 2 (for the case λ=1 ) and Lemma 1. Proceeding from (21), we obtain:

$$\begin{array}{c} a_{4}\;\leq \;\frac{1}{2\left(3+\delta \right)}\left[\beta_{1}\left|p_{3}-p_{1}p_{2}+\frac{p_{1}^{3}}{4}\right|+\left|\beta_{2}\right|\left|p_{1}\right|\left|p_{2}-\frac{p_{1}^{2}}{2}\right|+\frac{\left|\beta_{3}\right|}{4}{\left|p_{1}\right|}^{3}\right]+\frac{\left(1-\delta \right)\left(2-\delta \right)\beta_{1}^{3}{\left|p_{1}\right|}^{3}}{48{\left(1+\delta \right)}^{3}}\\ +\frac{\left(1-\delta \right)\beta_{1}\left|p_{1}\right|}{2\left(1+\delta \right)}\left[\frac{\beta_{1}\left|p_{2}\right|}{2\left(2+\delta \right)}+\left(\frac{\left|\beta_{2}\right|+\beta_{1}}{4\left(2+\delta \right)}+\frac{\left(1-\delta \right)\beta_{1}^{2}}{8{\left(1+\delta \right)}^{2}}\right){\left|p_{1}\right|}^{2}\right]. \end{array}$$

Applying Lemma 3 with parameters $u=\frac{1}{4},v=w=1$ , followed by Lemma 2 with λ=1 , and finally Lemma 1, yields the result stated in (15). Concluding this argument, Equation (22) provides:

$$a_{5}\;\leq \;\frac{\beta_{1}}{2\left(4+\delta \right)}\left(\left|p_{4}-p_{1}p_{3}\right|-\frac{{\left|p_{2}\right|}^{2}}{2}+\frac{3}{4}{\left|p_{1}\right|}^{2}\left|p_{2}-\frac{1}{3}\frac{p_{1}^{2}}{2}\right|\right)+\frac{\left|\beta_{2}\right|\left|p_{1}\right|}{2\left(4+\delta \right)}\left|p_{3}-p_{1}p_{2}+\frac{p_{1}^{3}}{4}\right|+\frac{\left|\beta_{2}\right|}{4\left(4+\delta \right)}{\left|p_{2}-\frac{p_{1}^{2}}{2}\right|}^{2}+\frac{3\left|\beta_{3}\right|{\left|p_{1}\right|}^{2}}{8\left(4+\delta \right)}\left|p_{2}-\frac{p_{1}^{2}}{2}\right|+\frac{\left|\beta_{4}\right|{\left|p_{1}\right|}^{4}}{16\left(4+\delta \right)}+\frac{\left(1-\delta \right)\left(2-\delta \right)}{2}\left(\frac{\beta_{1}^{2}{\left|p_{1}\right|}^{2}}{4{\left(1+\delta \right)}^{2}}\right)\left(\frac{\beta_{1}\left|p_{2}\right|}{2\left(2+\delta \right)}+\left[\frac{\left|\beta_{2}\right|+\beta_{1}}{4\left(2+\delta \right)}+\frac{\left(1-\delta \right)\beta_{1}^{2}}{8{\left(1+\delta \right)}^{2}}\right]{\left|p_{1}\right|}^{2}\right)+\frac{\left(1-\delta \right)\left(2-\delta \right)\left(3-\delta \right)}{24}\left(\frac{\beta_{1}^{4}{\left|p_{1}\right|}^{4}}{16{\left(1+\delta \right)}^{4}}\right)+\left(1-\delta \right)\left(\frac{\beta_{1}\left|p_{1}\right|}{2\left(1+\delta \right)}\right)\left(\frac{1}{2\left(3+\delta \right)}\left[\beta_{1}\left|p_{3}-p_{1}p_{2}+\frac{p_{1}^{3}}{4}\right|+\left|\beta_{2}\right|\left|p_{1}\right|\left|p_{2}-\frac{p_{1}^{2}}{2}\right|+\frac{\left|\beta_{3}\right|}{4}{\left|p_{1}\right|}^{3}\right]+\frac{\left(1-\delta \right)\left(2-\delta \right)\beta_{1}^{3}{\left|p_{1}\right|}^{3}}{48{\left(1+\delta \right)}^{3}}+\frac{\left(1-\delta \right)\beta_{1}\left|p_{1}\right|}{2\left(1+\delta \right)}\left[\frac{\beta_{1}\left|p_{2}\right|}{2\left(2+\delta \right)}+\left(\frac{\left|\beta_{2}\right|+\beta_{1}}{4\left(2+\delta \right)}+\frac{\left(1-\delta \right)\beta_{1}^{2}}{8{\left(1+\delta \right)}^{2}}\right){\left|p_{1}\right|}^{2}\right]\right)+\frac{\left(1-\delta \right)}{2}{\left(\frac{\beta_{1}\left|p_{2}\right|}{2\left(2+\delta \right)}+\left[\frac{\left|\beta_{2}\right|+\beta_{1}}{4\left(2+\delta \right)}+\frac{\left(1-\delta \right)\beta_{1}^{2}}{8{\left(1+\delta \right)}^{2}}\right]{\left|p_{1}\right|}^{2}\right)}^{2}.$$

### 3.3 Fekete-Szegö estimates for the class Λ(δ,b)

A central problem in the study of coefficient estimates for functions f in the class S is the analysis of the Fekete-Szegö functional, defined as:

$$FS\left(\tau ,f\right)=\left|a_{3}-\tau a_{2}^{2}\right|.$$ (23)

In Geometric Function Theory (GFT), this functional, named for mathematicians Michael Fekete and Gábor Szegö, is a pivotal tool. Its historical significance stems from its role in refuting the Littlewood-Paley conjecture. Consequently, it has been the subject of extensive research for numerous subclasses of S , as documented in references like. ^14– 22^

Theorem 2. Let f∈Λ(δ,b) . Then

$$FS\left(\tau ,f\right)\;\leq \;\left\{ \begin{array}{ccc} \frac{\beta_{1}}{2+\delta }\left(\frac{4\left(1-\delta \right)\left(2+\delta \right)\beta_{1}}{\delta {\left(1+\delta \right)}^{2}}+\frac{\beta_{2}}{\beta_{1}}-\frac{\tau \left(2+\delta \right)\beta_{1}}{{\left(1+\delta \right)}^{2}}\right) & \text{when} & \tau \;\leq \;\varphi_{1},\\ \frac{\beta_{1}}{2+\delta } & \text{when} & \varphi_{1}\;\leq \;\tau \;\leq \;\varphi_{2},\\ \frac{-\beta_{1}}{2+\delta }\left(\frac{4\left(1-\delta \right)\left(2+\delta \right)\beta_{1}}{\delta {\left(1+\delta \right)}^{2}}+\frac{\beta_{2}}{\beta_{1}}-\frac{\tau \left(2+\delta \right)\beta_{1}}{{\left(1+\delta \right)}^{2}}\right) & \text{when} & \tau \;\geq \;\varphi_{2},\\ \frac{\beta_{1}}{2+\delta }\max\left\{1,\varphi_{3}\right\} & \text{when} & \tau \;\in \;C, \end{array}\right.$$

where

$$\varphi_{1}=\frac{4\left(1-\delta \right)}{\delta }+\frac{\beta_{2}{\left(1+\delta \right)}^{2}}{\beta_{1}^{2}\left(2+\delta \right)}-\frac{{\left(1+\delta \right)}^{2}}{\beta_{1}\left(2+\delta \right)},$$

$$\varphi_{2}=\frac{4\left(1-\delta \right)}{\delta }+\frac{\beta_{2}{\left(1+\delta \right)}^{2}}{\beta_{1}^{2}\left(2+\delta \right)}+\frac{{\left(1+\delta \right)}^{2}}{\beta_{1}\left(2+\delta \right)}.$$

And

$$\varphi_{3}=\left|1-\lambda \right|=\left|\frac{4\left(1-\delta \right)\left(2+\delta \right)\beta_{1}}{\delta {\left(1+\delta \right)}^{2}}+\frac{\beta_{2}}{\beta_{1}}-\frac{\tau \left(2+\delta \right)\beta_{1}}{{\left(1+\delta \right)}^{2}}\right|.$$ (24)

Proof. Substituting Equations (19) and (20) into the functional defined in (23) yields

$$\begin{array}{c} a_{3}-\tau a_{2}^{2}=\frac{\beta_{1}p_{2}}{2\left(2+\delta \right)}+\left[\frac{\beta_{2}-\beta_{1}}{4\left(2+\delta \right)}+\frac{\left(1-\delta \right)\beta_{1}^{2}}{8{\left(1+\delta \right)}^{2}}\right]p_{1}^{2}-\tau {\left(\frac{\beta_{1}p_{1}}{2\left(1+\delta \right)}\right)}^{2}\\ =\frac{\beta_{1}}{2\left(2+\delta \right)}\left(p_{2}-\left[\frac{\tau \left(2+\delta \right)\beta_{1}}{{\left(1+\delta \right)}^{2}}-\frac{4\left(1-\delta \right)\left(2+\delta \right)\beta_{1}}{\delta {\left(1+\delta \right)}^{2}}-\frac{\beta_{2}}{\beta_{1}}+1\right]\frac{p_{1}^{2}}{2}\right). \end{array}$$

So that

$$FS\left(\tau ,f\right)=\left|a_{3}-\tau a_{2}^{2}\right|=\left|\frac{\beta_{1}}{2\left(2+\delta \right)}\left(p_{2}-\left[\frac{\tau \left(2+\delta \right)\beta_{1}}{{\left(1+\delta \right)}^{2}}-\frac{4\left(1-\delta \right)\left(2+\delta \right)\beta_{1}}{\delta {\left(1+\delta \right)}^{2}}-\frac{\beta_{2}}{\beta_{1}}+1\right]\frac{p_{1}^{2}}{2}\right)\right|.$$ (25)

If we set

$$\lambda =\frac{\tau \left(2+\delta \right)\beta_{1}}{{\left(1+\delta \right)}^{2}}-\frac{4\left(1-\delta \right)\left(2+\delta \right)\beta_{1}}{\delta {\left(1+\delta \right)}^{2}}-\frac{\beta_{2}}{\beta_{1}}+1.$$

An application of Lemma 2 to the expression in (25) leads to

$$\left|p_{2}-\lambda \frac{p_{1}^{2}}{2}\right|\;\leq \;2\left(1-\lambda \right)=2\left(\frac{4\left(1-\delta \right)\left(2+\delta \right)\beta_{1}}{\delta {\left(1+\delta \right)}^{2}}+\frac{\beta_{2}}{\beta_{1}}-\frac{\tau \left(2+\delta \right)\beta_{1}}{{\left(1+\delta \right)}^{2}}\right).$$ (26)

So that when λ≤0, we have

$$\tau \;\leq \;\frac{4\left(1-\delta \right)}{\delta }+\frac{\beta_{2}{\left(1+\delta \right)}^{2}}{\beta_{1}^{2}\left(2+\delta \right)}-\frac{{\left(1+\delta \right)}^{2}}{\beta_{1}\left(2+\delta \right)}.$$ (27)

Secondly,

$$\left|p_{2}-\lambda \frac{p_{1}^{2}}{2}\right|\;\leq \;2.$$ (28)

So that when 0≤λ≤2, we have

$$\frac{4\left(1-\delta \right)}{\delta }+\frac{\beta_{2}{\left(1+\delta \right)}^{2}}{\beta_{1}^{2}\left(2+\delta \right)}-\frac{{\left(1+\delta \right)}^{2}}{\beta_{1}\left(2+\delta \right)}\;\leq \;\tau \;\leq \;\frac{4\left(1-\delta \right)}{\delta }+\frac{\beta_{2}{\left(1+\delta \right)}^{2}}{\beta_{1}^{2}\left(2+\delta \right)}+\frac{{\left(1+\delta \right)}^{2}}{\beta_{1}\left(2+\delta \right)}.$$ (29)

Thirdly,

$$\left|p_{2}-\lambda \frac{p_{1}^{2}}{2}\right|\;\leq \;2\left(\lambda -1\right)=-2\left(\frac{4\left(1-\delta \right)\left(2+\delta \right)\beta_{1}}{\delta {\left(1+\delta \right)}^{2}}+\frac{\beta_{2}}{\beta_{1}}-\frac{\tau \left(2+\delta \right)\beta_{1}}{{\left(1+\delta \right)}^{2}}\right).$$ (30)

So that when λ≥2, we have

$$\tau \;\geq \;\frac{4\left(1-\delta \right)}{\delta }+\frac{\beta_{2}{\left(1+\delta \right)}^{2}}{\beta_{1}^{2}\left(2+\delta \right)}+\frac{{\left(1+\delta \right)}^{2}}{\beta_{1}\left(2+\delta \right)}.$$ (31)

Finally, if λ∈ℂ , then

$$1-\lambda =\frac{4\left(1-\delta \right)\left(2+\delta \right)\beta_{1}}{\delta {\left(1+\delta \right)}^{2}}+\frac{\beta_{2}}{\beta_{1}}-\frac{\tau \left(2+\delta \right)\beta_{1}}{{\left(1+\delta \right)}^{2}}.$$ (32)

Thus, the form of $\varphi_{3}$ in (24) is verified. Substituting Equations (26) through (32) into (25) completes the proof of the Theorem.

### 3.4 Hankel determinant estimates for the class Λ(δ,b)

The Hankel matrix, characterized by constant entries along each ascending skew-diagonal, was first introduced by the German mathematician Hermann Hankel (1839–1873) in the mid-nineteenth century. Hankel’s initial research applied this matrix to the analysis of number sequences and their determinants. Since then, its utility has expanded significantly, with applications now encompassing factorial fractions, ^23^ orthogonal polynomials, ^24^ power series with integer coefficients, ^25^ and the asymptotic properties of the determinants themselves. ^26^ Within Geometric Function Theory (GFT), Pommerenke ^27^ defined the Hankel determinant as follows:

$$H_{i,j}\left(f\right)=\left[\begin{array}{ccc} \begin{array}{c} a_{j}\\ a_{j+1} \end{array} & \begin{array}{c} a_{j+1}\\ \ldots \end{array} & \begin{array}{c} \ldots \\ \ldots \end{array}\;\begin{array}{c} a_{j+i-1}\\ \ldots \end{array}\\ \vdots & \vdots & \;\begin{array}{cc} \vdots & \vdots \end{array}\;\\ a_{j+i-1} & \ldots & \begin{array}{cc} \ldots & a_{j+2\left(i-1\right)} \end{array} \end{array}\right].$$ (33)

Here, i,j≥1 , and the entries $a_{j}$ represent the coefficients of functions f∈S . Pommerenke originally applied these Hankel determinants to analyze the singularities of complex functions.

Building upon Pommerenke’s foundation, ^27^ in a generalization of the Hankel determinant, Babalola ^28^ introduced a Fekete-Szegö parameter τ>0 into the structure of $H_{i,j}\left(f\right)$ , which led to the definition of the following determinants:

$$H_{i,j}^{\tau }\left(f\right)=\left[\begin{array}{ccc} \begin{array}{c} a_{j}\\ a_{j+1} \end{array} & \begin{array}{c} a_{j+1}\\ \ldots \end{array} & \begin{array}{cc} \begin{array}{c} \ldots \\ \ldots \end{array} & \begin{array}{c} {\mathrm{\tau a}}_{j+i-1}\\ \ldots \end{array} \end{array}\\ \vdots & \vdots & \;\begin{array}{cc} \vdots & \vdots \end{array}\;\\ a_{j+i-1} & \ldots & \begin{array}{cc} \ldots & a_{j+2\left(i-1\right)} \end{array} \end{array}\right].$$ (34)

Note that (34) simplifies to

$$\left|H_{2,1}^{\tau }\left(f\right)\right|=\left|a_{3}-\tau a_{2}^{2}\right|.$$ (35)

$$\left|H_{2,2}^{\tau }\left(f\right)\right|=\left|a_{2}a_{4}-\tau a_{3}^{2}\right|.$$ (36)

And

$$\left|H_{3,1}^{\tau }\left(f\right)\right|\;\leq \;\left|a_{3}\right|\left|a_{2}a_{4}-\tau a_{3}^{2}\right|+\left|a_{4}\right|\left|a_{2}a_{3}-\tau a_{4}\right|+\left|a_{5}\right|\left|a_{3}-\tau a_{2}^{2}\right|.$$ (37)

$$\left|H_{3,1}^{\tau }\left(f\right)\right|\;\leq \;\left|a_{3}\right|\left|H_{2,2}^{\tau }\left(f\right)\right|+\left|a_{4}\right|\left|L_{2}^{\tau }\left(f\right)\right|+\left|a_{5}\right|\left|H_{2,1}^{\tau }\left(f\right)\right|.$$ (38)

Where

$$\left|L_{j}^{\tau }\left(f\right)\right|=\left|a_{j}a_{j+1}-\tau a_{j-2}\right|\;\left(j\;\in \;\left\{2,3,4,\ldots \right\}\right).$$ (39)

Remark 2. The following observations are noted:

1. When τ=1 is substituted into (34), the modified determinant reduces to the original form, i.e., $H_{i,j}^{\tau }\left(f\right)=H_{i,j}\left(f\right)$ as defined in (33).
2. The absolute value of the second-order determinant $\left|H_{2,1}^{\tau }\left(f\right)\right|$ is equivalent to the Fekete-Szegö functional FS(τ,f) given in (23).

A comprehensive discussion of these properties can be found in the references. ^3, 20, 22, 28^ This study aims to determine the sharp upper bounds for the Hankel determinants in (36) and (38), treating the parameter τ as a positive real value.

Theorem 3. Let f∈Λ(δ,b) . Then

$$\left|H_{2,2}^{\tau }\left(f\right)\right|\;\leq \;\frac{\beta_{1}}{1+\delta }\left(\frac{\beta_{1}+2\left|\beta_{2}\right|+\left|\beta_{3}\right|}{3+\delta }+\frac{\left(1-\delta \right)\left(2-\delta \right)\beta_{1}^{3}}{6{\left(1+\delta \right)}^{3}}+\frac{\left(1-\delta \right)\beta_{1}^{2}}{\left(1+\delta \right)\left(2+\delta \right)}+\frac{\left(\left(1-\delta \right)\beta_{1}\right)\left(\left|\beta_{2}\right|+\beta_{1}\right)}{\left(1+\delta \right)\left(2+\delta \right)}+\frac{{\left(1-\delta \right)}^{2}\beta_{1}^{3}}{2{\left(1+\delta \right)}^{3}}\right)+\tau \left(\frac{2\beta_{1}+\left|\beta_{2}\right|}{2+\delta }+\frac{\left(1-\delta \right)\beta_{1}^{2}}{{\left(1+\delta \right)}^{2}}\right).$$

Proof. Substituting the expressions from (19), (20), and (21) into Equation (36) yields

$$a_{2}a_{4}-\tau a_{3}^{2}=\left(\frac{\beta_{1}p_{1}}{2\left(1+\delta \right)}\right)\left(\frac{1}{2\left(3+\delta \right)}\left[\beta_{1}\left(p_{3}-p_{1}p_{2}+\frac{p_{1}^{3}}{4}\right)+\beta_{2}p_{1}\left(p_{2}-\frac{p_{1}^{2}}{2}\right)+\frac{\beta_{3}}{4}p_{1}^{3}\right]-\frac{\left(1-\delta \right)\left(2-\delta \right)\beta_{1}^{3}p_{1}^{3}}{48{\left(1+\delta \right)}^{3}}+\frac{\left(1-\delta \right)\beta_{1}p_{1}}{2\left(1+\delta \right)}\left[\frac{\beta_{1}p_{2}}{2\left(2+\delta \right)}+\left(\frac{\beta_{2}-\beta_{1}}{4\left(2+\delta \right)}+\frac{\left(1-\delta \right)\beta_{1}^{2}}{8{\left(1+\delta \right)}^{2}}\right)p_{1}^{2}\right]\right)-\tau {\left(\frac{\beta_{1}p_{2}}{2\left(2+\delta \right)}+\left[\frac{\beta_{2}-\beta_{1}}{4\left(2+\delta \right)}+\frac{\left(1-\delta \right)\beta_{1}^{2}}{8{\left(1+\delta \right)}^{2}}\right]p_{1}^{2}\right)}^{2}.$$

Such that

$$\left|a_{2}a_{4}-\tau a_{3}^{2}\right|\;\leq \;\left(\frac{\beta_{1}}{2\left(1+\delta \right)}\left|p_{1}\right|\right)\left(\frac{1}{2\left(3+\delta \right)}\left[\beta_{1}\left|p_{3}-p_{1}p_{2}+\frac{p_{1}^{3}}{4}\right|+\left|\beta_{2}\right|\left|p_{1}\right|\left|p_{2}-\frac{p_{1}^{2}}{2}\right|+\frac{\left|\beta_{3}\right|}{4}{\left|p_{1}\right|}^{3}\right]+\frac{\left(1-\delta \right)\left(2-\delta \right)\beta_{1}^{3}{\left|p_{1}\right|}^{3}}{48{\left(1+\delta \right)}^{3}}+\frac{\left(1-\delta \right)\beta_{1}\left|p_{1}\right|}{2\left(1+\delta \right)}\left[\frac{\beta_{1}\left|p_{2}\right|}{2\left(2+\delta \right)}+\left(\frac{\left|\beta_{2}\right|+\beta_{1}}{4\left(2+\delta \right)}+\frac{\left(1-\delta \right)\beta_{1}^{2}}{8{\left(1+\delta \right)}^{2}}\right){\left|p_{1}\right|}^{2}\right]\right)+\tau {\left(\frac{\beta_{1}\left|p_{2}\right|}{2\left(2+\delta \right)}+\left[\frac{\left|\beta_{2}\right|+\beta_{1}}{4\left(2+\delta \right)}+\frac{\left(1-\delta \right)\beta_{1}^{2}}{8{\left(1+\delta \right)}^{2}}\right]{\left|p_{1}\right|}^{2}\right)}^{2}.$$

The desired result of the theorem is then obtained through the systematic application of Lemma 1, Lemma 3 (with parameters $u=\frac{1}{4},v=w=1$ ), and Lemma 2 (with λ=1 ).

Theorem 4. Let f∈Λ(δ,b). Then

$$\begin{array}{c} \left|L_{2}^{\tau }\left(f\right)\right|\;\leq \;\frac{2\beta_{1}^{2}+\beta_{1}\left|\beta_{2}\right|}{\left(1+\delta \right)\left(2+\delta \right)}+\frac{\left(1-\delta \right)\beta_{1}^{3}}{2{\left(1+\delta \right)}^{3}}\\ +\tau \left(\frac{\beta_{1}+2\left|\beta_{2}\right|+\left|\beta_{3}\right|}{3+\delta }+\frac{\left(1-\delta \right)\left(2-\delta \right)\beta_{1}^{3}}{6{\left(1+\delta \right)}^{3}}+\frac{\left(1-\delta \right)\beta_{1}^{2}}{\left(1+\delta \right)\left(2+\delta \right)}+\frac{\left(\left(1-\delta \right)\beta_{1}\right)\left(\left|\beta_{2}\right|+\beta_{1}\right)}{\left(1+\delta \right)\left(2+\delta \right)}+\frac{{\left(1-\delta \right)}^{2}\beta_{1}^{3}}{2{\left(1+\delta \right)}^{3}}\right). \end{array}$$

Proof. By substituting Equations (19), (20), and (21) into (39), we find that

$$a_{2}a_{3}-\tau a_{4}=\left(\frac{\beta_{1}p_{1}}{2\left(1+\delta \right)}\right)\left(\frac{\beta_{1}p_{2}}{2\left(2+\delta \right)}+\left[\frac{\beta_{2}-\beta_{1}}{4\left(2+\delta \right)}+\frac{\left(1-\delta \right)\beta_{1}^{2}}{8{\left(1+\delta \right)}^{2}}\right]p_{1}^{2}\right)-\tau \left(\frac{1}{2\left(3+\delta \right)}\left[\beta_{1}\left(p_{3}-p_{1}p_{2}+\frac{p_{1}^{3}}{4}\right)+\beta_{2}p_{1}\left(p_{2}-\frac{p_{1}^{2}}{2}\right)+\frac{\beta_{3}}{4}p_{1}^{3}\right]-\frac{\left(1-\delta \right)\left(2-\delta \right)\beta_{1}^{3}p_{1}^{3}}{48{\left(1+\delta \right)}^{3}}+\frac{\left(1-\delta \right)\beta_{1}p_{1}}{2\left(1+\delta \right)}\left[\frac{\beta_{1}p_{2}}{2\left(2+\delta \right)}+\left(\frac{\beta_{2}-\beta_{1}}{4\left(2+\delta \right)}+\frac{\left(1-\delta \right)\beta_{1}^{2}}{8{\left(1+\delta \right)}^{2}}\right)p_{1}^{2}\right]\right).$$

Therefore,

$$\left|a_{2}a_{3}-\tau a_{4}\right|\;\leq \;\left(\frac{\beta_{1}}{2\left(1+\delta \right)}\left|p_{1}\right|\right)\left(\frac{\beta_{1}\left|p_{2}\right|}{2\left(2+\delta \right)}+\left[\frac{\left|\beta_{2}\right|+\beta_{1}}{4\left(2+\delta \right)}+\frac{\left(1-\delta \right)\beta_{1}^{2}}{8{\left(1+\delta \right)}^{2}}\right]{\left|p_{1}\right|}^{2}\right)+\tau \left(\frac{1}{2\left(3+\delta \right)}\left[\beta_{1}\left|p_{3}-p_{1}p_{2}+\frac{p_{1}^{3}}{4}\right|+\left|\beta_{2}\right|\left|p_{1}\right|\left|p_{2}-\frac{p_{1}^{2}}{2}\right|+\frac{\left|\beta_{3}\right|}{4}{\left|p_{1}\right|}^{3}\right]+\frac{\left(1-\delta \right)\left(2-\delta \right)\beta_{1}^{3}{\left|p_{1}\right|}^{3}}{48{\left(1+\delta \right)}^{3}}+\frac{\left(1-\delta \right)\beta_{1}\left|p_{1}\right|}{2\left(1+\delta \right)}\left[\frac{\beta_{1}\left|p_{2}\right|}{2\left(2+\delta \right)}+\left(\frac{\left|\beta_{2}\right|+\beta_{1}}{4\left(2+\delta \right)}+\frac{\left(1-\delta \right)\beta_{1}^{2}}{8{\left(1+\delta \right)}^{2}}\right){\left|p_{1}\right|}^{2}\right]\right).$$

The result of the theorem is then obtained by applying Lemma 3 (with $u=\frac{1}{4},$ v=w=1 ), Lemma 2 (with λ=1 ), and Lemma 1.

Theorem 5. Let f∈Λ(δ,b). Then

$$\left|H_{3,1}^{\tau }\left(f\right)\right|\;\leq \;\left(\frac{2\beta_{1}+\left|\beta_{2}\right|}{2+\delta }+\frac{\left(1-\delta \right)\beta_{1}^{2}}{{\left(1+\delta \right)}^{2}}\right)\times \left\{\frac{\beta_{1}}{1+\delta }\left(\frac{\beta_{1}+2\left|\beta_{2}\right|+\left|\beta_{3}\right|}{3+\delta }+\frac{\left(1-\delta \right)\left(2-\delta \right)\beta_{1}^{3}}{6{\left(1+\delta \right)}^{3}}+\frac{\left(1-\delta \right)\beta_{1}^{2}}{\left(1+\delta \right)\left(2+\delta \right)}+\frac{\left(\left(1-\delta \right)\beta_{1}\right)\left(\left|\beta_{2}\right|+\beta_{1}\right)}{\left(1+\delta \right)\left(2+\delta \right)}+\frac{{\left(1-\delta \right)}^{2}\beta_{1}^{3}}{2{\left(1+\delta \right)}^{3}}\right)+\tau \left(\frac{2\beta_{1}+\left|\beta_{2}\right|}{2+\delta }+\frac{\left(1-\delta \right)\beta_{1}^{2}}{{\left(1+\delta \right)}^{2}}\right)\right\}+\left(\frac{\beta_{1}+2\left|\beta_{2}\right|+\left|\beta_{3}\right|}{3+\delta }+\frac{\left(1-\delta \right)\left(2-\delta \right)\beta_{1}^{3}}{6{\left(1+\delta \right)}^{3}}+\frac{\left(1-\delta \right)\beta_{1}^{2}}{\left(1+\delta \right)\left(2+\delta \right)}+\frac{\left(\left(1-\delta \right)\beta_{1}\right)\left(\left|\beta_{2}\right|+\beta_{1}\right)}{\left(1+\delta \right)\left(2+\delta \right)}+\frac{{\left(1-\delta \right)}^{2}\beta_{1}^{3}}{2{\left(1+\delta \right)}^{3}}\right)\left\{\frac{2\beta_{1}^{2}+\beta_{1}\left|\beta_{2}\right|}{\left(1+\delta \right)\left(2+\delta \right)}+\frac{\left(1-\delta \right)\beta_{1}^{3}}{2{\left(1+\delta \right)}^{3}}+\tau \left(\frac{\beta_{1}+2\left|\beta_{2}\right|+\left|\beta_{3}\right|}{3+\delta }+\frac{\left(1-\delta \right)\left(2-\delta \right)\beta_{1}^{3}}{6{\left(1+\delta \right)}^{3}}+\frac{\left(1-\delta \right)\beta_{1}^{2}}{\left(1+\delta \right)\left(2+\delta \right)}+\frac{\left(\left(1-\delta \right)\beta_{1}\right)\left(\left|\beta_{2}\right|+\beta_{1}\right)}{\left(1+\delta \right)\left(2+\delta \right)}+\frac{{\left(1-\delta \right)}^{2}\beta_{1}^{3}}{2{\left(1+\delta \right)}^{3}}\right)\right\}+\left(\frac{5\beta_{1}+3\left|\beta_{2}\right|+3\left|\beta_{3}\right|+\left|\beta_{4}\right|}{\left(4+\delta \right)}+\frac{\left(1-\delta \right)\left(2-\delta \right)}{2}\left(\frac{\beta_{1}^{2}}{{\left(1+\delta \right)}^{2}}\right)\left(\frac{2\beta_{1}+\left|\beta_{2}\right|}{\left(2+\delta \right)}+\frac{\left(1-\delta \right)\beta_{1}^{2}}{2{\left(1+\delta \right)}^{2}}\right)+\frac{\left(1-\delta \right)\left(2-\delta \right)\left(3-\delta \right)\beta_{1}^{4}}{24{\left(1+\delta \right)}^{4}}+\frac{\left(1-\delta \right)\beta_{1}}{\left(1+\delta \right)}\left(\frac{\beta_{1}+2\left|\beta_{2}\right|+\left|\beta_{3}\right|}{3+\delta }+\frac{\left(1-\delta \right)\left(2-\delta \right)\beta_{1}^{3}}{6{\left(1+\delta \right)}^{3}}+\frac{\left(1-\delta \right)\beta_{1}^{2}}{\left(1+\delta \right)\left(2+\delta \right)}+\frac{\left(\left(1-\delta \right)\beta_{1}\right)\left(\left|\beta_{2}\right|+\beta_{1}\right)}{\left(1+\delta \right)\left(2+\delta \right)}+\frac{{\left(1-\delta \right)}^{2}\beta_{1}^{3}}{2{\left(1+\delta \right)}^{3}}\right)+\frac{\left(1-\delta \right)}{2}{\left(\frac{2\beta_{1}+\left|\beta_{2}\right|}{2+\delta }+\frac{\left(1-\delta \right)\beta_{1}^{2}}{{\left(1+\delta \right)}^{2}}\right)}^{2}\right)\left\{\frac{\beta_{1}}{2+\delta }\right\}.$$

Proof. The result is established by applying the findings of Theorems 1, 2, 3, and 4 to Equation (38), followed by the necessary algebraic simplifications.

## 4. Conclusion

This study investigated the properties of a new class of functions that generalizes Yamaguchi and starlike functions, both of which hold significant importance within the well-known class of Bazilevič functions. The new class, Λ(δ,b) , building upon the Ma-Minda function, a new class of functions is formulated based on fundamental tenets of Geometric Function Theory (GFT). Key results derived for this class include tight upper estimates on the initial coefficients, the Fekete-Szegö functional for parameters, and the second and third-order Hankel determinants involving a parameter τ>0 .

A notable feature of the newly defined set is its generality; it reduces to several well-known and previously studied function classes when specific parameters are chosen within their declared intervals.

## Ethical considerations

The study did not involve human participants or animals.

## Data Availability

No data are associated with this article.
