# Bounds on Hankel Determinants with Fekete-Szegö Parameter for Bazilević Functions

**DOI:** 10.12688/f1000research.173313.2

**Published:** 2026-04-16

**Authors:** Nathir Khaled, Abdul Rahman S.Juma

**Affiliations:** 1 Mathematics, University of Anbar, Ramadi, Al Anbar Governorate, Iraq

**Keywords:** Hankel determinant, Bazileviˇc function, subordination, Yamaguchi function, Ma-Minda function, Fekete-Szegö estimate

## Abstract

**Background:**

In Geometric Function Theory, a central area of complex analysis, researchers study the geometric properties of analytic and univalent functions in the unit disk. A significant part of this work involves defining some subclasses of functions and investigating their properties, such as coefficient estimates and Hankel determinants, which reveal important geometric information. This paper introduces a comprehensive subclass of analytic-univalent functions that generalizes well-known families like Yamaguchi and starlike functions within the broader Bazilević framework.

**Methods and Results:**

Using the theory of Ma-Minda functions and the principle of subordination, sharp bounds for the initial coefficients, the Fekete-Szegö functionals with parameters, and the Hankel determinants are established for this subclass. The results are proven to be sharp, meaning they are the best possible. Furthermore, it is shown that this general class reduces to several previously known function families for specific parameter values, demonstrating its wide applicability.

**Conclusions:**

This research successfully defines and analyzes a comprehensive subclass of analytic and univalent functions. The obtained sharp bounds for coefficient-related problems generalize and extend existing results in the literature. The work provides a unified framework for studying various function families, contributing valuable insights and tools for further exploration in Geometric Function Theory.

## 1. Introduction

Geometric function theory (GFT) is a significant branch of complex analysis concerned with the geometric properties of analytic functions. Its applications extend to various mathematical and physical disciplines, including *q*-calculus, special functions, orthogonal polynomials, and conformal mappings.

Let A be the family of analytic functions on the open unit disk Ω={z∈ℂ:|z|<1} , normalized by the conditions f(0)=0 and $f^{\prime }\left(0\right)=1$ . Such functions have the series expansion:

$$f\left(z\right)=z+\sum_{j=2}^{\infty }a_{j}z^{j},$$

the subclass S⊂A consists of all univalent (one-to-one) functions in Ω .

A cornerstone of GFT is the coefficient problem, which seeks to determine the possible values of the coefficients $a_{j}$ and the bounds of functionals constructed from them. This problem was famously initiated by Bieberbach’s 1916 conjecture that | $a_{j}$ | ≤j for all j≥2 . The eventual proof of this conjecture by de Branges in 1985 ^1^ underscored the profound depth of this area of research. The pursuit of sharp bounds for other coefficient functionals, such as the Fekete-Szegö functional and Hankel determinants, remains an active and central theme in GFT.

Prominent subclasses of S , including starlike, convex, close-to-convex, and Yamaguchi functions, are defined based on the geometric characteristics of their image domains. The class of Bazilević functions, introduced in, ^2^ represents one of the largest known subclasses of S .

A powerful tool in defining these classes is the principle of subordination (denoted f≺F ). For two functions ( f,F∈A ), we say f is subordinate to F if there exists an analytic function:

$$s\left(z\right)=s_{1}z+s_{2}z^{2}+s_{3}z^{3}\ldots$$

with s(0)=0 and |s(z)|<1 such that f(z)=F(s(z)). If F is univalent, this is equivalent to f(0)=F(0) and f ( Ω ) ⊂ F(Ω) .

The Carathéodory class *P* consists of functions p(z) analytic in *Ω* with *p*(0) = 1 and Re(p(z))>0 . Such functions have the series expansion:

In a significant unification effort, Ma and Minda ^3^ introduced a general class using a function b(z) that is analytic, univalent, has a positive real part, and is characterized by a Taylor series $b\left(z\right)=1+B_{1}z+B_{2}z^{2}+\ldots$ with $B_{1}$ > 0. This framework elegantly encapsulates many previously studied classes.

A canonical and extremal function within the class P is the Möbius function:

$$m_{0}\left(z\right)=\frac{1+z}{1-z}=1+2\sum_{j=1}^{\infty }z^{j}\;\left(z\;\in \;\Omega \right),$$

there exists a fundamental relationship between Carathéodory functions p(z) and functions s(z) analytic in Ω with |s(z)|<1 and s(0)=0 . This relationship is given by the following transformation:

$$p\left(z\right)=\frac{1+s\left(z\right)}{1-s\left(z\right)}⟺s\left(z\right)=\frac{p\left(z\right)-1}{p\left(z\right)+1}\;\left(z\;\in \;\Omega \right),$$

substituting the series expansion for p(z) into this relation yields the series expansion for s(z) :

$$s\left(z\right)=\frac{1}{2}\left[p_{1}z+\left(p_{2}-\frac{p_{1}^{2}}{2}\right)z^{2}+\left(p_{3}-p_{1}p_{2}+\frac{p_{1}^{3}}{4}\right)z^{3}+\ldots \right].$$

A significant unification of several subclasses of starlike and convex functions was achieved by Ma and Minda ^3^ in 1994. They introduced a function b(z) , which is analytic and univalent with a series expansion of the form:

$$b\left(z\right)=1+\beta_{1}z+\beta_{2}z^{2}+\beta_{3}z^{3}+\ldots \left(\beta_{1}>0,\beta_{k}\;\in \;\mathbb{R},z\;\in \;\Omega \right).$$ (1)

This function *b* ( *z*) has the following key properties:

( Re(b(z)))>0.

b(0)=1 .

$b^{\prime }\left(0\right)>0$ .

*b* ( *z*) maps the unit disk Ω onto a domain that is starlike with respect to 1 and symmetric about the real axis.

By composing this function with s(z) , we obtain:

$$\begin{array}{c} b\left(s\left(z\right)\right)=1+\beta_{1}s\left(z\right)+\beta_{2}{\left(s\left(z\right)\right)}^{2}+\beta_{3}{\left(s\left(z\right)\right)}^{3}+\ldots \\ =1+\frac{\beta_{1}}{2}p_{1}z+\left[\frac{\beta_{1}}{2}\left(p_{2}-\frac{p_{1}^{2}}{2}\right)+\frac{\beta_{2}}{4}p_{1}^{2}\right]\;z^{2}\\ +\left[\frac{\beta_{1}}{2}\left(p_{3}-p_{1}p_{2}+\frac{p_{1}^{3}}{4}\right)+\frac{\beta_{2}}{2}p_{1}\left(p_{2}-\frac{p_{1}^{2}}{2}\right)+\frac{\beta_{3}}{8}p_{1}^{3}\right]z^{3}+\ldots \end{array}$$ (2)

If a function f∈A maps the unit disk Ω onto a starlike domain, then f is classified as a starlike function. This geometric property is characterized by the analytic condition:

$$f\;\in \;\mathrm{ST}=Re\left(\frac{zf^{\prime }\left(z\right)}{f\left(z\right)}\right)>0,\;z\;\in \;\Omega \;.$$ (3)

The extremal function for the class ST is the Koebe function:

$$k\left(z\right)=\frac{z}{{\left(1-z\right)}^{2}}.$$

The class ST was first presented via Alexander, ^4^ though its corresponding geometric property was actually characterized earlier via Nevanlinna in 1921. ^5^ Research into starlike functions has since expanded significantly, leading to diverse formulations and applications, as explored by authors such as Lasode and Opoola. ^6^ Later, in 1956, Yamaguchi ^7^ defined a specific subclass of S characterized by the following condition:

$$f\;\in \;\Upsilon =Re\left(f^{\prime }\left(z\right)\right)>0,z\;\in \;\Omega \;.$$ (4)

Various properties of functions in the Yamaguchi class Υ -such as univalence, radii problems, partial sums, and growth, distortion, and inclusion theorems-have been demonstrated in the literature on GFT. ^7– 9^ The class of Bazilević functions ^2^ is one of the largest subclasses of S . Singh ^10^ introduced important subclasses characterized by:

$$Re\frac{zf^{\prime }\left(z\right)f{\left(z\right)}^{\delta -1}}{h{\left(z\right)}^{\delta }}>0\;\text{and}\;Re\frac{zf^{\prime }\left(z\right)f{\left(z\right)}^{\delta -1}}{z^{\delta }}>0.$$ (5)

with *δ* > 0.

In this paper, we build upon these foundations to define a new and comprehensive subclass Λ( *δ, b*) of analytic-univalent functions using a combination of concepts from Bazilević, Yamaguchi, and starlike functions, subordinated to a Ma-Minda function. We then proceed to derive sharp coefficient bounds, Fekete-Szegö inequalities, and Hankel determinant estimates for functions belonging to this class, demonstrating that it generalizes several important families of functions previously studied that have been mentioned by Tang H. and et al. ^11^ and Lasode A. O. and et al. ^12^ in the literature.

## 2. Lemmas

The following lemmas for Carathéodory functions are essential in proving our main results.

Lemma 1. ^5^Let p∈P . Then

$$\left|p_{j}\right|\;≦\;2\forall j\;\in \;\left\{1,2,3,4,\ldots \right\}\;\text{and}\;|c_{2}-\frac{c_{1}^{2}}{2}|\;\leq \;2-\frac{|c_{1}{|}^{2}}{2}.$$

Lemma 2. ^13^Let p∈P . Then

$$\left|p_{2}-\lambda \frac{p_{1}^{2}}{2}\right|\;≦\;\left\{ \begin{array}{ccc} 2\left(1-\lambda \right) & \text{when} & \lambda \;≦\;0\\ 2 & \text{when} & 0\;≦\;\lambda \;≦\;2\\ 2\left(\lambda -1\right) & \text{when} & \lambda \;≦\;2\\ 2\max\left\{1,|1-\lambda |\right\} & \text{when} & \lambda \;\in \;\mathbb{C}\; \end{array}\right.$$

Lemma 3. ^14^Let p∈P . Then for any real numbers u,v and w,

$$\left|up_{1}^{3}-vp_{1}p_{2}+wp_{3}\right|\;≦\;2\left|u\right|+2\left|v-2u\right|+2\left|u-v+w\right|.$$

Lemma 4. ^15^Let p∈P . Then for i,j∈{1,2,3,…},

$$\left|p_{i+j}-\mu p_{i}p_{j}\right|\;≦\;\left\{ \begin{array}{cc} 2 & \text{when}\;0\;≦\;\mu \;≦\;1\\ 2\left|2\mu -1\right| & \text{elsewhere} \end{array}\right..$$

## 3. Main results

### 3.1 A new class of analytic-univalent functions

We now introduce a new class of analytic-univalent functions that generalizes the Bazilevič family.

Definition 1. The class Λ(δ,b) consists of all functions f∈A satisfying

$${\left(f^{\prime }\left(z\right)\right)}^{\delta }{\left(\frac{zf^{\prime }\left(z\right)}{f\left(z\right)}\right)}^{1-\delta }≺\;b\left(z\right),$$ (6)

with 0≤δ≤1 and where b(z) is the Ma-Minda function defined in ( 1).

Remark 1. The class Λ(δ,b) generalizes several well-known families:

1. When δ=1 , condition ( 6) reduces to $f^{\prime }(z)\;≺\;b(z)$ . In particular, $b(z)=m_{0}(z)$ , we obtain the Yamaguchi class Υ defined in ( 4).
2. When δ=0 , condition ( 5) reduces to $\frac{zf^{\prime }(z)}{f(z)}\;≺\;b(z)$ . If $b(z)=m_{0}(z)$ , we obtain the starlike class ST defined in ( 3).

For general δ and b(z), this class provides a unified framework for studying Bazilević-type functions with Ma–Minda subordination.

### 3.2 Coefficient estimates for set Λ(δ,b)

Theorem 1. If a function f∈A belongs to the class Λ(δ,b) , then

$$\left|a_{2}\right|\;\leq \;\frac{\beta_{1}}{1+\delta }$$ (7)

$$\left|a_{3}\right|\;\leq \;\frac{2\beta_{1}+\left|\beta_{2}\right|}{2+\delta }+\frac{\left(1-\delta \right)\beta_{1}^{2}}{{\left(1+\delta \right)}^{2}}$$ (8)

$$\left|a_{4}\right|\;\leq \;\frac{\beta_{1}+2\left|\beta_{2}\right|+\left|\beta_{3}\right|}{3+\delta }+\frac{\left(1-\delta \right)\left(2-\delta \right)\beta_{1}^{3}}{6{\left(1+\delta \right)}^{3}}+\frac{\left(1-\delta \right)\beta_{1}^{2}}{\left(1+\delta \right)\left(2+\delta \right)}+\frac{\left(\left(1-\delta \right)\beta_{1}\right)\left(\left|\beta_{2}\right|+\beta_{1}\right)}{\left(1+\delta \right)\left(2+\delta \right)}+\frac{{\left(1-\delta \right)}^{2}\beta_{1}^{3}}{2{\left(1+\delta \right)}^{3}}.$$ (9)

And

$$\begin{array}{c} \left|a_{5}\right|\;\leq \;\frac{5\beta_{1}+3\left|\beta_{2}\right|+3\left|\beta_{3}\right|+\left|\beta_{4}\right|}{\left(4+\delta \right)}+\frac{\left(1-\delta \right)\left(2-\delta \right)}{2}\left(\frac{\beta_{1}^{2}}{{\left(1+\delta \right)}^{2}}\right)\left(\frac{2\beta_{1}+\left|\beta_{2}\right|}{\left(2+\delta \right)}+\frac{\left(1-\delta \right)\beta_{1}^{2}}{2{\left(1+\delta \right)}^{2}}\right)+\frac{\left(1-\delta \right)\left(2-\delta \right)\left(3-\delta \right)\beta_{1}^{4}}{24{\left(1+\delta \right)}^{4}}\\ +\frac{\left(1-\delta \right)\beta_{1}}{\left(1+\delta \right)}\left(\frac{\beta_{1}+2\left|\beta_{2}\right|+\left|\beta_{3}\right|}{3+\delta }+\frac{\left(1-\delta \right)\left(2-\delta \right)\beta_{1}^{3}}{6{\left(1+\delta \right)}^{3}}+\frac{\left(1-\delta \right)\beta_{1}^{2}}{\left(1+\delta \right)\left(2+\delta \right)}+\frac{\left(\left(1-\delta \right)\beta_{1}\right)\left(\left|\beta_{2}\right|+\beta_{1}\right)}{\left(1+\delta \right)\left(2+\delta \right)}+\frac{{\left(1-\delta \right)}^{2}\beta_{1}^{3}}{2{\left(1+\delta \right)}^{3}}\right)\\ +\frac{\left(1-\delta \right)}{2}{\left(\frac{2\beta_{1}+\left|\beta_{2}\right|}{2+\delta }+\frac{\left(1-\delta \right)\beta_{1}^{2}}{{\left(1+\delta \right)}^{2}}\right)}^{2}. \end{array}$$

Proof. Let f∈A be a member of Λ(δ,b) . By the definition of subordination, there exists a Schwarz function such

$$\left(f^{\prime }\left(z\right)\right){\left(\frac{zf^{\prime }\left(z\right)}{f\left(z\right)}\right)}^{1-\delta }=b\left(s\left(z\right)\right)\;\left(z\;\in \;\Omega \right),$$ (10)

using the binomial theorem, the left-hand side of ( 10) can be expressed as the series:

$$\begin{array}{c} {\left(f^{\prime }\left(z\right)\right)}^{\delta }{\left(\frac{zf^{\prime }\left(z\right)}{f\left(z\right)}\right)}^{1-\delta }=1+\left(1+\delta \right)a_{2}z+\frac{1}{2}\left(2+\delta \right)\left[2a_{3}-\left(1-\delta \right)a_{2}^{2}\right]z^{2}\\ +\frac{\left(3+\delta \right)}{6}\left[\left(1-\delta \right)\left(2-\delta \right)a_{2}^{3}-6\left(1-\delta \right)a_{2}a_{3}+6a_{4}\right]z^{3}+\ldots , \end{array}$$ (11)

consequently, a comparison of the coefficients in ( 11) and ( 2) gives

$$a_{2}=\frac{\beta_{1}p_{1}}{2\left(1+\delta \right)},$$ (12)

$$a_{3}=\frac{\beta_{1}p_{2}}{2\left(2+\delta \right)}+\left[\frac{\beta_{2}-\beta_{1}}{4\left(2+\delta \right)}+\frac{\left(1-\delta \right)\beta_{1}^{2}}{8{\left(1+\delta \right)}^{2}}\right]p_{1}^{2},$$ (13)

$$\begin{array}{c} a_{4}=\frac{1}{2\left(3+\delta \right)}\left[\beta_{1}\left(p_{3}-p_{1}p_{2}+\frac{p_{1}^{3}}{4}\right)+\beta_{2}p_{1}\left(p_{2}-\frac{p_{1}^{2}}{2}\right)+\frac{\beta_{3}}{4}p_{1}^{3}\right]-\frac{\left(1-\delta \right)\left(2-\delta \right)\beta_{1}^{3}p_{1}^{3}}{48{\left(1+\delta \right)}^{3}}\\ +\frac{\left(1-\delta \right)\beta_{1}p_{1}}{2\left(1+\delta \right)}\left[\frac{\beta_{1}p_{2}}{2\left(2+\delta \right)}+\left(\frac{\beta_{2}-\beta_{1}}{4\left(2+\delta \right)}+\frac{\left(1-\delta \right)\beta_{1}^{2}}{8{\left(1+\delta \right)}^{2}}\right)p_{1}^{2}\right]. \end{array}$$ (14)

And

$$a_{5}=\frac{\beta_{1}}{2\left(4+\delta \right)}\left(p_{4}-p_{1}p_{3}-\frac{p_{2}^{2}}{2}+\frac{3p_{1}^{2}p_{2}}{4}-\frac{p_{1}^{4}}{8}\right)+\frac{\beta_{2}p_{1}}{2\left(4+\delta \right)}\left(p_{3}-p_{1}p_{2}+\frac{p_{1}^{3}}{4}\right)+\frac{\beta_{2}}{4\left(4+\delta \right)}{\left(p_{2}-\frac{p_{1}^{2}}{2}\right)}^{2}+\frac{3\beta_{3}p_{1}^{2}}{8\left(4+\delta \right)}\left(p_{2}-\frac{p_{1}^{2}}{2}\right)+\frac{\beta_{4}p_{1}^{4}}{16\left(4+\delta \right)}-\frac{\left(1-\delta \right)\left(2-\delta \right)}{2}{\left(\frac{\beta_{1}p_{1}}{2\left(1+\delta \right)}\right)}^{2}\left(\frac{\beta_{1}p_{2}}{2\left(2+\delta \right)}+\left[\frac{\beta_{2}-\beta_{1}}{4\left(2+\delta \right)}+\frac{\left(1-\delta \right)\beta_{1}^{2}}{8{\left(1+\delta \right)}^{2}}\right]p_{1}^{2}\right)+\frac{\left(1-\delta \right)\left(2-\delta \right)\left(3-\delta \right)}{24}{\left(\frac{\beta_{1}p_{1}}{2\left(1+\delta \right)}\right)}^{4}+\left(1-\delta \right)\left(\frac{\beta_{1}p_{1}}{2\left(1+\delta \right)}\right)\left(\frac{1}{2\left(3+\delta \right)}\left[\beta_{1}\left(p_{3}-p_{1}p_{2}+\frac{p_{1}^{3}}{4}\right)+\beta_{2}p_{1}\left(p_{2}-\frac{p_{1}^{2}}{2}\right)+\frac{\beta_{3}}{4}p_{1}^{3}\right]-\frac{\left(1-\delta \right)\left(2-\delta \right)\beta_{1}^{3}p_{1}^{3}}{48{\left(1+\delta \right)}^{3}}+\frac{\left(1-\delta \right)\beta_{1}p_{1}}{2\left(1+\delta \right)}\left[\frac{\beta_{1}p_{2}}{2\left(2+\delta \right)}+\left(\frac{\beta_{2}-\beta_{1}}{4\left(2+\delta \right)}+\frac{\left(1-\delta \right)\beta_{1}^{2}}{8{\left(1+\delta \right)}^{2}}\right)p_{1}^{2}\right]\right)+\frac{\left(1-\delta \right)}{2}{\left(\frac{\beta_{1}p_{2}}{2\left(2+\delta \right)}+\left[\frac{\beta_{2}-\beta_{1}}{4\left(2+\delta \right)}+\frac{\left(1-\delta \right)\beta_{1}^{2}}{8{\left(1+\delta \right)}^{2}}\right]p_{1}^{2}\right)}^{2}.$$ (15)

Apparently, ( 12) gives

$$\left|a_{2}\right|=\frac{\beta_{1}}{2\left(1+\delta \right)}\left|p_{1}\right|.$$

Utilizing Lemma 1, we obtain the result given in ( 7). Additionally, from ( 13) it follows that

$$a_{3}\;\leq \;\frac{\beta_{1}\left|p_{2}\right|}{2\left(2+\delta \right)}+\left[\frac{\left|\beta_{2}\right|+\beta_{1}}{4\left(2+\delta \right)}+\frac{\left(1-\delta \right)\beta_{1}^{2}}{8{\left(1+\delta \right)}^{2}}\right]{\left|p_{1}\right|}^{2}.$$

The result in ( 8) follows from an application of Lemma 2 (for the case λ=1 ) and Lemma 1. Proceeding from ( 14), we obtain:

$$\begin{array}{c} a_{4}\;\leq \;\frac{1}{2\left(3+\delta \right)}\left[\beta_{1}\left|p_{3}-p_{1}p_{2}+\frac{p_{1}^{3}}{4}\right|+\left|\beta_{2}\right|\left|p_{1}\right|\left|p_{2}-\frac{p_{1}^{2}}{2}\right|+\frac{\left|\beta_{3}\right|}{4}{\left|p_{1}\right|}^{3}\right]+\frac{\left(1-\delta \right)\left(2-\delta \right)\beta_{1}^{3}{\left|p_{1}\right|}^{3}}{48{\left(1+\delta \right)}^{3}}\\ +\frac{\left(1-\delta \right)\beta_{1}\left|p_{1}\right|}{2\left(1+\delta \right)}\left[\frac{\beta_{1}\left|p_{2}\right|}{2\left(2+\delta \right)}+\left(\frac{\left|\beta_{2}\right|+\beta_{1}}{4\left(2+\delta \right)}+\frac{\left(1-\delta \right)\beta_{1}^{2}}{8{\left(1+\delta \right)}^{2}}\right){\left|p_{1}\right|}^{2}\right]. \end{array}$$

Applying Lemma 3 with parameters $u=\frac{1}{4},v=w=1$ , followed by Lemma 2 with λ=1 , and finally Lemma 1, yields the result stated in ( 9). Concluding this argument, Equation (15) provides:

$$a_{5}\;\leq \;\frac{\beta_{1}}{2\left(4+\delta \right)}\left(\left|p_{4}-p_{1}p_{3}\right|-\frac{{\left|p_{2}\right|}^{2}}{2}+\frac{3}{4}{\left|p_{1}\right|}^{2}\left|p_{2}-\frac{1}{3}\frac{p_{1}^{2}}{2}\right|\right)+\frac{\left|\beta_{2}\right|\left|p_{1}\right|}{2\left(4+\delta \right)}\left|p_{3}-p_{1}p_{2}+\frac{p_{1}^{3}}{4}\right|+\frac{\left|\beta_{2}\right|}{4\left(4+\delta \right)}{\left|p_{2}-\frac{p_{1}^{2}}{2}\right|}^{2}+\frac{3\left|\beta_{3}\right|{\left|p_{1}\right|}^{2}}{8\left(4+\delta \right)}\left|p_{2}-\frac{p_{1}^{2}}{2}\right|+\frac{\left|\beta_{4}\right|{\left|p_{1}\right|}^{4}}{16\left(4+\delta \right)}+\frac{\left(1-\delta \right)\left(2-\delta \right)}{2}\left(\frac{\beta_{1}^{2}{\left|p_{1}\right|}^{2}}{4{\left(1+\delta \right)}^{2}}\right)\left(\frac{\beta_{1}\left|p_{2}\right|}{2\left(2+\delta \right)}+\left[\frac{\left|\beta_{2}\right|+\beta_{1}}{4\left(2+\delta \right)}+\frac{\left(1-\delta \right)\beta_{1}^{2}}{8{\left(1+\delta \right)}^{2}}\right]{\left|p_{1}\right|}^{2}\right)+\frac{\left(1-\delta \right)\left(2-\delta \right)\left(3-\delta \right)}{24}\left(\frac{\beta_{1}^{4}{\left|p_{1}\right|}^{4}}{16{\left(1+\delta \right)}^{4}}\right)+\left(1-\delta \right)\left(\frac{\beta_{1}\left|p_{1}\right|}{2\left(1+\delta \right)}\right)\left(\frac{1}{2\left(3+\delta \right)}\left[\beta_{1}\left|p_{3}-p_{1}p_{2}+\frac{p_{1}^{3}}{4}\right|+\left|\beta_{2}\right|\left|p_{1}\right|\left|p_{2}-\frac{p_{1}^{2}}{2}\right|+\frac{\left|\beta_{3}\right|}{4}{\left|p_{1}\right|}^{3}\right]+\frac{\left(1-\delta \right)\left(2-\delta \right)\beta_{1}^{3}{\left|p_{1}\right|}^{3}}{48{\left(1+\delta \right)}^{3}}+\frac{\left(1-\delta \right)\beta_{1}\left|p_{1}\right|}{2\left(1+\delta \right)}\left[\frac{\beta_{1}\left|p_{2}\right|}{2\left(2+\delta \right)}+\left(\frac{\left|\beta_{2}\right|+\beta_{1}}{4\left(2+\delta \right)}+\frac{\left(1-\delta \right)\beta_{1}^{2}}{8{\left(1+\delta \right)}^{2}}\right){\left|p_{1}\right|}^{2}\right]\right)+\frac{\left(1-\delta \right)}{2}{\left(\frac{\beta_{1}\left|p_{2}\right|}{2\left(2+\delta \right)}+\left[\frac{\left|\beta_{2}\right|+\beta_{1}}{4\left(2+\delta \right)}+\frac{\left(1-\delta \right)\beta_{1}^{2}}{8{\left(1+\delta \right)}^{2}}\right]{\left|p_{1}\right|}^{2}\right)}^{2}.$$

### 3.3 Fekete-Szegö estimates for the class Λ(δ,b)

A central problem in the study of coefficient estimates for functions f in the class S is the analysis of the Fekete-Szegö functional, defined as:

$$FS\left(\tau ,f\right)=\left|a_{3}-\tau a_{2}^{2}\right|.$$ (16)

In Geometric Function Theory (GFT), this functional, named for mathematicians Michael Fekete and Gábor Szegö, is a pivotal tool. Its historical significance stems from its role in refuting the Littlewood-Paley conjecture. Consequently, it has been the subject of extensive research for numerous subclasses of S , as documented in references like. ^16– 24^

Theorem 2. Let f∈Λ(δ,b) . Then

$$FS\left(\tau ,f\right)\;\leq \;\left\{ \begin{array}{ccc} \frac{\beta_{1}}{2+\delta }\left(\frac{4\left(1-\delta \right)\left(2+\delta \right)\beta_{1}}{\delta {\left(1+\delta \right)}^{2}}+\frac{\beta_{2}}{\beta_{1}}-\frac{\tau \left(2+\delta \right)\beta_{1}}{{\left(1+\delta \right)}^{2}}\right) & \text{when} & \tau \;\leq \;\varphi_{1},\\ \frac{\beta_{1}}{2+\delta } & \text{when} & \varphi_{1}\;\leq \;\tau \;\leq \;\varphi_{2},\\ \frac{-\beta_{1}}{2+\delta }\left(\frac{4\left(1-\delta \right)\left(2+\delta \right)\beta_{1}}{\delta {\left(1+\delta \right)}^{2}}+\frac{\beta_{2}}{\beta_{1}}-\frac{\tau \left(2+\delta \right)\beta_{1}}{{\left(1+\delta \right)}^{2}}\right) & \text{when} & \tau \;\geq \;\varphi_{2},\\ \frac{\beta_{1}}{2+\delta }\max\left\{1,\varphi_{3}\right\} & \text{when} & \tau \;\in \;C, \end{array}\right.$$

where

$$\varphi_{1}=\frac{4\left(1-\delta \right)}{\delta }+\frac{\beta_{2}{\left(1+\delta \right)}^{2}}{\beta_{1}^{2}\left(2+\delta \right)}-\frac{{\left(1+\delta \right)}^{2}}{\beta_{1}\left(2+\delta \right)},$$

$$\varphi_{2}=\frac{4\left(1-\delta \right)}{\delta }+\frac{\beta_{2}{\left(1+\delta \right)}^{2}}{\beta_{1}^{2}\left(2+\delta \right)}+\frac{{\left(1+\delta \right)}^{2}}{\beta_{1}\left(2+\delta \right)}.$$

And

$$\varphi_{3}=\left|1-\lambda \right|=\left|\frac{4\left(1-\delta \right)\left(2+\delta \right)\beta_{1}}{\delta {\left(1+\delta \right)}^{2}}+\frac{\beta_{2}}{\beta_{1}}-\frac{\tau \left(2+\delta \right)\beta_{1}}{{\left(1+\delta \right)}^{2}}\right|.$$ (17)

Proof. Substituting Equations (12) and (13) into the functional defined in ( 16) yields

$$\begin{array}{c} a_{3}-\tau a_{2}^{2}=\frac{\beta_{1}p_{2}}{2\left(2+\delta \right)}+\left[\frac{\beta_{2}-\beta_{1}}{4\left(2+\delta \right)}+\frac{\left(1-\delta \right)\beta_{1}^{2}}{8{\left(1+\delta \right)}^{2}}\right]p_{1}^{2}-\tau {\left(\frac{\beta_{1}p_{1}}{2\left(1+\delta \right)}\right)}^{2}\\ =\frac{\beta_{1}}{2\left(2+\delta \right)}\left(p_{2}-\left[\frac{\tau \left(2+\delta \right)\beta_{1}}{{\left(1+\delta \right)}^{2}}-\frac{4\left(1-\delta \right)\left(2+\delta \right)\beta_{1}}{\delta {\left(1+\delta \right)}^{2}}-\frac{\beta_{2}}{\beta_{1}}+1\right]\frac{p_{1}^{2}}{2}\right). \end{array}$$

So that

$$FS\left(\tau ,f\right)=\left|a_{3}-\tau a_{2}^{2}\right|=\left|\frac{\beta_{1}}{2\left(2+\delta \right)}\left(p_{2}-\left[\frac{\tau \left(2+\delta \right)\beta_{1}}{{\left(1+\delta \right)}^{2}}-\frac{4\left(1-\delta \right)\left(2+\delta \right)\beta_{1}}{\delta {\left(1+\delta \right)}^{2}}-\frac{\beta_{2}}{\beta_{1}}+1\right]\frac{p_{1}^{2}}{2}\right)\right|.$$ (18)

If we set

$$\lambda =\frac{\tau \left(2+\delta \right)\beta_{1}}{{\left(1+\delta \right)}^{2}}-\frac{4\left(1-\delta \right)\left(2+\delta \right)\beta_{1}}{\delta {\left(1+\delta \right)}^{2}}-\frac{\beta_{2}}{\beta_{1}}+1.$$

An application of Lemma 2 to the expression in ( 18) leads to

$$\left|p_{2}-\lambda \frac{p_{1}^{2}}{2}\right|\;\leq \;2\left(1-\lambda \right)=2\left(\frac{4\left(1-\delta \right)\left(2+\delta \right)\beta_{1}}{\delta {\left(1+\delta \right)}^{2}}+\frac{\beta_{2}}{\beta_{1}}-\frac{\tau \left(2+\delta \right)\beta_{1}}{{\left(1+\delta \right)}^{2}}\right).$$ (19)

So that when λ≤0, we have

$$\tau \;\leq \;\frac{4\left(1-\delta \right)}{\delta }+\frac{\beta_{2}{\left(1+\delta \right)}^{2}}{\beta_{1}^{2}\left(2+\delta \right)}-\frac{{\left(1+\delta \right)}^{2}}{\beta_{1}\left(2+\delta \right)}.$$

Secondly,

$$\left|p_{2}-\lambda \frac{p_{1}^{2}}{2}\right|\;\leq \;2.$$

So that when 0≤λ≤2, we have

$$\frac{4\left(1-\delta \right)}{\delta }+\frac{\beta_{2}{\left(1+\delta \right)}^{2}}{\beta_{1}^{2}\left(2+\delta \right)}-\frac{{\left(1+\delta \right)}^{2}}{\beta_{1}\left(2+\delta \right)}\;\leq \;\tau \;\leq \;\frac{4\left(1-\delta \right)}{\delta }+\frac{\beta_{2}{\left(1+\delta \right)}^{2}}{\beta_{1}^{2}\left(2+\delta \right)}+\frac{{\left(1+\delta \right)}^{2}}{\beta_{1}\left(2+\delta \right)}.$$

Thirdly,

$$\left|p_{2}-\lambda \frac{p_{1}^{2}}{2}\right|\;\leq \;2\left(\lambda -1\right)=-2\left(\frac{4\left(1-\delta \right)\left(2+\delta \right)\beta_{1}}{\delta {\left(1+\delta \right)}^{2}}+\frac{\beta_{2}}{\beta_{1}}-\frac{\tau \left(2+\delta \right)\beta_{1}}{{\left(1+\delta \right)}^{2}}\right).$$

So that when λ≥2, we have

$$\tau \;\geq \;\frac{4\left(1-\delta \right)}{\delta }+\frac{\beta_{2}{\left(1+\delta \right)}^{2}}{\beta_{1}^{2}\left(2+\delta \right)}+\frac{{\left(1+\delta \right)}^{2}}{\beta_{1}\left(2+\delta \right)}.$$

Finally, if λ∈ℂ , then

$$1-\lambda =\frac{4\left(1-\delta \right)\left(2+\delta \right)\beta_{1}}{\delta {\left(1+\delta \right)}^{2}}+\frac{\beta_{2}}{\beta_{1}}-\frac{\tau \left(2+\delta \right)\beta_{1}}{{\left(1+\delta \right)}^{2}}.$$ (20)

Thus, the form of $\varphi_{3}$ in ( 17) is verified. Substituting Equations (19) through (20) into (18) completes the proof of the Theorem.

### 3.4 Hankel determinant estimates for the class Λ(δ,b)

The Hankel matrix, characterized by constant entries along each ascending skew-diagonal, was first introduced by the German mathematician Hermann Hankel (1839–1873) in the mid-nineteenth century. Hankel’s initial research applied this matrix to the analysis of number sequences and their determinants. Since then, its utility has expanded significantly, with applications now encompassing factorial fractions, ^25^ orthogonal polynomials, ^26^ power series with integer coefficients, ^27^ and the asymptotic properties of the determinants themselves. ^28^ Within Geometric Function Theory (GFT), Pommerenke ^29^ defined the Hankel determinant as follows:

$$H_{i,j}\left(f\right)=\left[\begin{array}{ccc} \begin{array}{c} a_{j}\\ a_{j+1} \end{array} & \begin{array}{c} a_{j+1}\\ \ldots \end{array} & \begin{array}{c} \ldots \\ \ldots \end{array}\;\begin{array}{c} a_{j+i-1}\\ \ldots \end{array}\\ \vdots & \vdots & \;\begin{array}{cc} \vdots & \vdots \end{array}\;\\ a_{j+i-1} & \ldots & \begin{array}{cc} \ldots & a_{j+2\left(i-1\right)} \end{array} \end{array}\right].$$ (21)

Here, i,j≥1 , and the entries $a_{j}$ represent the coefficients of functions f∈A . Pommerenke originally applied these Hankel determinants to analyze the singularities of complex functions.

Building upon Pommerenke’s foundation, ^29^ in a generalization of the Hankel determinant, Babalola ^30^ introduced a Fekete-Szegö parameter τ>0 into the structure of $H_{i,j}\left(f\right)$ , which led to the definition of the following determinants:

$$H_{i,j}^{\tau }\left(f\right)=\left[\begin{array}{ccc} \begin{array}{c} a_{j}\\ a_{j+1} \end{array} & \begin{array}{c} a_{j+1}\\ \ldots \end{array} & \begin{array}{cc} \begin{array}{c} \ldots \\ \ldots \end{array} & \begin{array}{c} {\mathrm{\tau a}}_{j+i-1}\\ \ldots \end{array} \end{array}\\ \vdots & \vdots & \;\begin{array}{cc} \vdots & \vdots \end{array}\;\\ a_{j+i-1} & \ldots & \begin{array}{cc} \ldots & a_{j+2\left(i-1\right)} \end{array} \end{array}\right].$$ (22)

Note that ( 22) simplifies to

$$\left|H_{2,1}^{\tau }\left(f\right)\right|=\left|a_{3}-\tau a_{2}^{2}\right|.$$ (23)

The functional $H_{2,1}^{\tau }(f)$ is commonly referred to as the Fekete–Szegő functional ^31– 33^

$$\left|H_{2,2}^{\tau }\left(f\right)\right|=\left|a_{2}a_{4}-\tau a_{3}^{2}\right|.$$ (24)

Recent research has focused extensively on determining upper bounds for the second-order HD $H_{2,2}^{\tau }(f)$ for various subclasses of analytic functions, as demonstrated. ^34– 36^

Also

$$\left|H_{3,1}^{\tau }\left(f\right)\right|\;\leq \;\left|a_{3}\right|\left|a_{2}a_{4}-\tau a_{3}^{2}\right|+\left|a_{4}\right|\left|a_{2}a_{3}-\tau a_{4}\right|+\left|a_{5}\right|\left|a_{3}-\tau a_{2}^{2}\right|.$$

$$\left|H_{3,1}^{\tau }\left(f\right)\right|\;\leq \;\left|a_{3}\right|\left|H_{2,2}^{\tau }\left(f\right)\right|+\left|a_{4}\right|\left|L_{2}^{\tau }\left(f\right)\right|+\left|a_{5}\right|\left|H_{2,1}^{\tau }\left(f\right)\right|.$$ (25)

Where

$$\left|L_{j}^{\tau }\left(f\right)\right|=\left|a_{j}a_{j+1}-\tau a_{j-2}\right|\;\left(j\;\in \;\left\{2,3,4,\ldots \right\}\right).$$ (26)

Related studies have also addressed third and fourth-order HDs. ^37– 41^

Remark 2. The following observations are noted:

1. When τ=1 is substituted into ( 22), the modified determinant reduces to the original form, i.e., $H_{i,j}^{\tau }\left(f\right)=H_{i,j}\left(f\right)$ as defined in ( 21).
2. The absolute value of the second-order determinant $\left|H_{2,1}^{\tau }\left(f\right)\right|$ in ( 23) is equivalent to the Fekete-Szegö functional FS(τ,f) given in ( 16).

A comprehensive discussion of these properties can be found in the references. ^3, 22, 24, 30^ This study aims to determine the sharp upper bounds for the Hankel determinants in ( 24) and ( 25), treating the parameter τ as a positive real value.

Theorem 3. Let f∈Λ(δ,b) . Then

$$\left|H_{2,2}^{\tau }\left(f\right)\right|\;\leq \;\frac{\beta_{1}}{1+\delta }\left(\frac{\beta_{1}+2\left|\beta_{2}\right|+\left|\beta_{3}\right|}{3+\delta }+\frac{\left(1-\delta \right)\left(2-\delta \right)\beta_{1}^{3}}{6{\left(1+\delta \right)}^{3}}+\frac{\left(1-\delta \right)\beta_{1}^{2}}{\left(1+\delta \right)\left(2+\delta \right)}+\frac{\left(\left(1-\delta \right)\beta_{1}\right)\left(\left|\beta_{2}\right|+\beta_{1}\right)}{\left(1+\delta \right)\left(2+\delta \right)}+\frac{{\left(1-\delta \right)}^{2}\beta_{1}^{3}}{2{\left(1+\delta \right)}^{3}}\right)+\tau \left(\frac{2\beta_{1}+\left|\beta_{2}\right|}{2+\delta }+\frac{\left(1-\delta \right)\beta_{1}^{2}}{{\left(1+\delta \right)}^{2}}\right).$$

Proof. Substituting the expressions from ( 12), ( 13), and ( 14) into Equation (24) yields

$$a_{2}a_{4}-\tau a_{3}^{2}=\left(\frac{\beta_{1}p_{1}}{2\left(1+\delta \right)}\right)\left(\frac{1}{2\left(3+\delta \right)}\left[\beta_{1}\left(p_{3}-p_{1}p_{2}+\frac{p_{1}^{3}}{4}\right)+\beta_{2}p_{1}\left(p_{2}-\frac{p_{1}^{2}}{2}\right)+\frac{\beta_{3}}{4}p_{1}^{3}\right]-\frac{\left(1-\delta \right)\left(2-\delta \right)\beta_{1}^{3}p_{1}^{3}}{48{\left(1+\delta \right)}^{3}}+\frac{\left(1-\delta \right)\beta_{1}p_{1}}{2\left(1+\delta \right)}\left[\frac{\beta_{1}p_{2}}{2\left(2+\delta \right)}+\left(\frac{\beta_{2}-\beta_{1}}{4\left(2+\delta \right)}+\frac{\left(1-\delta \right)\beta_{1}^{2}}{8{\left(1+\delta \right)}^{2}}\right)p_{1}^{2}\right]\right)-\tau {\left(\frac{\beta_{1}p_{2}}{2\left(2+\delta \right)}+\left[\frac{\beta_{2}-\beta_{1}}{4\left(2+\delta \right)}+\frac{\left(1-\delta \right)\beta_{1}^{2}}{8{\left(1+\delta \right)}^{2}}\right]p_{1}^{2}\right)}^{2}.$$

Such that

$$\left|a_{2}a_{4}-\tau a_{3}^{2}\right|\;\leq \;\left(\frac{\beta_{1}}{2\left(1+\delta \right)}\left|p_{1}\right|\right)\left(\frac{1}{2\left(3+\delta \right)}\left[\beta_{1}\left|p_{3}-p_{1}p_{2}+\frac{p_{1}^{3}}{4}\right|+\left|\beta_{2}\right|\left|p_{1}\right|\left|p_{2}-\frac{p_{1}^{2}}{2}\right|+\frac{\left|\beta_{3}\right|}{4}{\left|p_{1}\right|}^{3}\right]+\frac{\left(1-\delta \right)\left(2-\delta \right)\beta_{1}^{3}{\left|p_{1}\right|}^{3}}{48{\left(1+\delta \right)}^{3}}+\frac{\left(1-\delta \right)\beta_{1}\left|p_{1}\right|}{2\left(1+\delta \right)}\left[\frac{\beta_{1}\left|p_{2}\right|}{2\left(2+\delta \right)}+\left(\frac{\left|\beta_{2}\right|+\beta_{1}}{4\left(2+\delta \right)}+\frac{\left(1-\delta \right)\beta_{1}^{2}}{8{\left(1+\delta \right)}^{2}}\right){\left|p_{1}\right|}^{2}\right]\right)+\tau {\left(\frac{\beta_{1}\left|p_{2}\right|}{2\left(2+\delta \right)}+\left[\frac{\left|\beta_{2}\right|+\beta_{1}}{4\left(2+\delta \right)}+\frac{\left(1-\delta \right)\beta_{1}^{2}}{8{\left(1+\delta \right)}^{2}}\right]{\left|p_{1}\right|}^{2}\right)}^{2}.$$

The desired result of the theorem is then obtained through the systematic application of Lemma 1, Lemma 3 (with parameters $u=\frac{1}{4},v=w=1$ ), and Lemma 2 (with λ=1 ).

Theorem 4. Let f∈Λ(δ,b). Then

$$\begin{array}{c} \left|L_{2}^{\tau }\left(f\right)\right|\;\leq \;\frac{2\beta_{1}^{2}+\beta_{1}\left|\beta_{2}\right|}{\left(1+\delta \right)\left(2+\delta \right)}+\frac{\left(1-\delta \right)\beta_{1}^{3}}{2{\left(1+\delta \right)}^{3}}\\ +\tau \left(\frac{\beta_{1}+2\left|\beta_{2}\right|+\left|\beta_{3}\right|}{3+\delta }+\frac{\left(1-\delta \right)\left(2-\delta \right)\beta_{1}^{3}}{6{\left(1+\delta \right)}^{3}}+\frac{\left(1-\delta \right)\beta_{1}^{2}}{\left(1+\delta \right)\left(2+\delta \right)}+\frac{\left(\left(1-\delta \right)\beta_{1}\right)\left(\left|\beta_{2}\right|+\beta_{1}\right)}{\left(1+\delta \right)\left(2+\delta \right)}+\frac{{\left(1-\delta \right)}^{2}\beta_{1}^{3}}{2{\left(1+\delta \right)}^{3}}\right). \end{array}$$

Proof. By substituting Equations (12), (13), and (14) into (26), we find that

$$a_{2}a_{3}-\tau a_{4}=\left(\frac{\beta_{1}p_{1}}{2\left(1+\delta \right)}\right)\left(\frac{\beta_{1}p_{2}}{2\left(2+\delta \right)}+\left[\frac{\beta_{2}-\beta_{1}}{4\left(2+\delta \right)}+\frac{\left(1-\delta \right)\beta_{1}^{2}}{8{\left(1+\delta \right)}^{2}}\right]p_{1}^{2}\right)-\tau \left(\frac{1}{2\left(3+\delta \right)}\left[\beta_{1}\left(p_{3}-p_{1}p_{2}+\frac{p_{1}^{3}}{4}\right)+\beta_{2}p_{1}\left(p_{2}-\frac{p_{1}^{2}}{2}\right)+\frac{\beta_{3}}{4}p_{1}^{3}\right]-\frac{\left(1-\delta \right)\left(2-\delta \right)\beta_{1}^{3}p_{1}^{3}}{48{\left(1+\delta \right)}^{3}}+\frac{\left(1-\delta \right)\beta_{1}p_{1}}{2\left(1+\delta \right)}\left[\frac{\beta_{1}p_{2}}{2\left(2+\delta \right)}+\left(\frac{\beta_{2}-\beta_{1}}{4\left(2+\delta \right)}+\frac{\left(1-\delta \right)\beta_{1}^{2}}{8{\left(1+\delta \right)}^{2}}\right)p_{1}^{2}\right]\right).$$

Therefore,

$$\left|a_{2}a_{3}-\tau a_{4}\right|\;\leq \;\left(\frac{\beta_{1}}{2\left(1+\delta \right)}\left|p_{1}\right|\right)\left(\frac{\beta_{1}\left|p_{2}\right|}{2\left(2+\delta \right)}+\left[\frac{\left|\beta_{2}\right|+\beta_{1}}{4\left(2+\delta \right)}+\frac{\left(1-\delta \right)\beta_{1}^{2}}{8{\left(1+\delta \right)}^{2}}\right]{\left|p_{1}\right|}^{2}\right)+\tau \left(\frac{1}{2\left(3+\delta \right)}\left[\beta_{1}\left|p_{3}-p_{1}p_{2}+\frac{p_{1}^{3}}{4}\right|+\left|\beta_{2}\right|\left|p_{1}\right|\left|p_{2}-\frac{p_{1}^{2}}{2}\right|+\frac{\left|\beta_{3}\right|}{4}{\left|p_{1}\right|}^{3}\right]+\frac{\left(1-\delta \right)\left(2-\delta \right)\beta_{1}^{3}{\left|p_{1}\right|}^{3}}{48{\left(1+\delta \right)}^{3}}+\frac{\left(1-\delta \right)\beta_{1}\left|p_{1}\right|}{2\left(1+\delta \right)}\left[\frac{\beta_{1}\left|p_{2}\right|}{2\left(2+\delta \right)}+\left(\frac{\left|\beta_{2}\right|+\beta_{1}}{4\left(2+\delta \right)}+\frac{\left(1-\delta \right)\beta_{1}^{2}}{8{\left(1+\delta \right)}^{2}}\right){\left|p_{1}\right|}^{2}\right]\right).$$

The result of the theorem is then obtained by applying Lemma 3 (with $u=\frac{1}{4},$ v=w=1 ), Lemma 2 (with λ=1 ), and Lemma 1.

Theorem 5. Let f∈Λ(δ,b). Then

$$\left|H_{3,1}^{\tau }\left(f\right)\right|\;\leq \;\left(\frac{2\beta_{1}+\left|\beta_{2}\right|}{2+\delta }+\frac{\left(1-\delta \right)\beta_{1}^{2}}{{\left(1+\delta \right)}^{2}}\right)\times \left\{\frac{\beta_{1}}{1+\delta }\left(\frac{\beta_{1}+2\left|\beta_{2}\right|+\left|\beta_{3}\right|}{3+\delta }+\frac{\left(1-\delta \right)\left(2-\delta \right)\beta_{1}^{3}}{6{\left(1+\delta \right)}^{3}}+\frac{\left(1-\delta \right)\beta_{1}^{2}}{\left(1+\delta \right)\left(2+\delta \right)}+\frac{\left(\left(1-\delta \right)\beta_{1}\right)\left(\left|\beta_{2}\right|+\beta_{1}\right)}{\left(1+\delta \right)\left(2+\delta \right)}+\frac{{\left(1-\delta \right)}^{2}\beta_{1}^{3}}{2{\left(1+\delta \right)}^{3}}\right)+\tau \left(\frac{2\beta_{1}+\left|\beta_{2}\right|}{2+\delta }+\frac{\left(1-\delta \right)\beta_{1}^{2}}{{\left(1+\delta \right)}^{2}}\right)\right\}+\left(\frac{\beta_{1}+2\left|\beta_{2}\right|+\left|\beta_{3}\right|}{3+\delta }+\frac{\left(1-\delta \right)\left(2-\delta \right)\beta_{1}^{3}}{6{\left(1+\delta \right)}^{3}}+\frac{\left(1-\delta \right)\beta_{1}^{2}}{\left(1+\delta \right)\left(2+\delta \right)}+\frac{\left(\left(1-\delta \right)\beta_{1}\right)\left(\left|\beta_{2}\right|+\beta_{1}\right)}{\left(1+\delta \right)\left(2+\delta \right)}+\frac{{\left(1-\delta \right)}^{2}\beta_{1}^{3}}{2{\left(1+\delta \right)}^{3}}\right)\left\{\frac{2\beta_{1}^{2}+\beta_{1}\left|\beta_{2}\right|}{\left(1+\delta \right)\left(2+\delta \right)}+\frac{\left(1-\delta \right)\beta_{1}^{3}}{2{\left(1+\delta \right)}^{3}}+\tau \left(\frac{\beta_{1}+2\left|\beta_{2}\right|+\left|\beta_{3}\right|}{3+\delta }+\frac{\left(1-\delta \right)\left(2-\delta \right)\beta_{1}^{3}}{6{\left(1+\delta \right)}^{3}}+\frac{\left(1-\delta \right)\beta_{1}^{2}}{\left(1+\delta \right)\left(2+\delta \right)}+\frac{\left(\left(1-\delta \right)\beta_{1}\right)\left(\left|\beta_{2}\right|+\beta_{1}\right)}{\left(1+\delta \right)\left(2+\delta \right)}+\frac{{\left(1-\delta \right)}^{2}\beta_{1}^{3}}{2{\left(1+\delta \right)}^{3}}\right)\right\}+\left(\frac{5\beta_{1}+3\left|\beta_{2}\right|+3\left|\beta_{3}\right|+\left|\beta_{4}\right|}{\left(4+\delta \right)}+\frac{\left(1-\delta \right)\left(2-\delta \right)}{2}\left(\frac{\beta_{1}^{2}}{{\left(1+\delta \right)}^{2}}\right)\left(\frac{2\beta_{1}+\left|\beta_{2}\right|}{\left(2+\delta \right)}+\frac{\left(1-\delta \right)\beta_{1}^{2}}{2{\left(1+\delta \right)}^{2}}\right)+\frac{\left(1-\delta \right)\left(2-\delta \right)\left(3-\delta \right)\beta_{1}^{4}}{24{\left(1+\delta \right)}^{4}}+\frac{\left(1-\delta \right)\beta_{1}}{\left(1+\delta \right)}\left(\frac{\beta_{1}+2\left|\beta_{2}\right|+\left|\beta_{3}\right|}{3+\delta }+\frac{\left(1-\delta \right)\left(2-\delta \right)\beta_{1}^{3}}{6{\left(1+\delta \right)}^{3}}+\frac{\left(1-\delta \right)\beta_{1}^{2}}{\left(1+\delta \right)\left(2+\delta \right)}+\frac{\left(\left(1-\delta \right)\beta_{1}\right)\left(\left|\beta_{2}\right|+\beta_{1}\right)}{\left(1+\delta \right)\left(2+\delta \right)}+\frac{{\left(1-\delta \right)}^{2}\beta_{1}^{3}}{2{\left(1+\delta \right)}^{3}}\right)+\frac{\left(1-\delta \right)}{2}{\left(\frac{2\beta_{1}+\left|\beta_{2}\right|}{2+\delta }+\frac{\left(1-\delta \right)\beta_{1}^{2}}{{\left(1+\delta \right)}^{2}}\right)}^{2}\right)\left\{\frac{\beta_{1}}{2+\delta }\right\}.$$

Proof. The result is established by applying the findings of Theorems 1, 2, 3, and 4 to Equation (25), followed by the necessary algebraic simplifications.

## 4. Conclusion

This study investigated the properties of a new class of functions that generalizes Yamaguchi and starlike functions, both of which hold significant importance within the well-known class of Bazilevič functions. The new class, Λ(δ,b) , building upon the Ma-Minda function, a new class of functions is formulated based on fundamental tenets of Geometric Function Theory (GFT). Key results derived for this class include tight upper estimates on the initial coefficients, the Fekete-Szegö functional for parameters, and the second and third-order Hankel determinants involving a parameter τ>0 .

A notable feature of the newly defined set is its generality; it reduces to several well-known and previously studied function classes when specific parameters are chosen within their declared intervals.

## Ethical considerations

The study did not involve human participants or animals.

## Data Availability

No data are associated with this article.
