# Supplementary material for: Bounds on Hankel Determinants with Fekete-Szegö Parameter for Bazilević Functions
Source: F1000Res. 2026 Apr 16;15:261. Originally published 2026 Feb 14. [Version 2] doi: 10.12688/f1000research.173313.2 (PMC13174934; doi:10.12688/f1000research.173313.2)
Supplement: Peer review history

# Reviewer report: Reviewer response for version 2

**DOI:** 10.5256/f1000research.198199.r475634

**Published:** 2026-05-05

**Authors:** Afis Saliu

**Affiliations:** 1 University of the Gambia, Serrekuna, The Gambia

The manuscript has shown some improvement; however, several important issues still need to be carefully addressed, as outlined below:

 \begin{itemize}

     \item[(1)] The abstract remains somewhat vague and does not adequately reflect the main contributions of the paper. It is recommended to revise it along the following lines: ``In this paper, we introduce a comprehensive subclass of analytic univalent functions that generalizes well-known families, including Yamaguchi and starlike functions, within the Bazilevic framework. For this class, we derive sharp coefficient bounds and establish estimates for the second and third Hankel determinants. Our results extend and unify several existing results in the literature, thereby providing a broader framework for the study of analytic-univalent functions.''

     \item[(2)] On page 3 of the introduction, it is stated that coefficient problems seek to determine the possible values of the coefficient $a_{j}$. This statement is inaccurate. Coefficient problems are primarily concerned with determining bounds for the coefficients rather than their exact values.

     \item[(3)] The series representation of the Carathéodory class is missing and should be included for completeness.

     \item[(4)] The properties of the Ma--Minda function $b(z)$, as mentioned toward the end of page 3, are incomplete. Furthermore, the term ``canonical function'' within the class $P$ is unclear and requires proper justification. It is advisable to remove this terminology unless a precise meaning is provided.

     \item[(5)] The concept of the Ma--Minda class is introduced more than once in the introduction. This redundancy should be eliminated by presenting the concept in a unified and coherent manner.

     \item[(6)] The authors claim on page 5 that the results obtained are sharp. However, none of the results are demonstrated to be sharp, nor are corresponding extremal functions provided. Given the nature of such results, it is essential to include extremal functions to justify the sharpness and validity of the bounds.

     \item[(7)] Several of the derived bounds appear excessively large, particularly those for $\lvert a_{4}\rvert$, $\lvert a_{5}\rvert$, $\lvert \mathcal{H}_{2, 2}(f)\rvert$, $\lvert \mathcal{L}_{2}(f)\rvert$, and $\lvert \mathcal{H}_{3, 1}(f)\rvert$. These estimates should be simplified where possible, presented more clearly, and supported by extremal functions to confirm their accuracy.

     \item[(8)] There is a bulk citation on page 7 (references 16--24). If these works are indeed central to the study, their specific contributions should be briefly discussed in relation to the present work.

     \item[(9)] A number of references appear to be irrelevant to the current study and should be carefully reviewed and removed where appropriate.

 \end{itemize}

 \begin{center}

     \section*{Recommendation}

 \end{center}

 In its present form, the manuscript requires major revision before it can be considered for indexing

Is the work clearly and accurately presented and does it cite the current literature?

No

If applicable, is the statistical analysis and its interpretation appropriate?

Not applicable

Are all the source data underlying the results available to ensure full reproducibility?

No source data required

Is the study design appropriate and is the work technically sound?

No

Are the conclusions drawn adequately supported by the results?

Partly

Are sufficient details of methods and analysis provided to allow replication by others?

Yes

Reviewer Expertise:

Complex Analysis (Geometric function theory)

I confirm that I have read this submission and believe that I have an appropriate level of expertise to state that I do not consider it to be of an acceptable scientific standard, for reasons outlined above.

# Reviewer report: Reviewer response for version 2

**DOI:** 10.5256/f1000research.198199.r475635

**Published:** 2026-04-17

**Authors:** suha jumaa hammad

**Affiliations:** 1 University of Tikrit, Tikrit, Iraq

The revised version reflects a clear and careful response to the points raised in the previous review.

 The presentation has been improved, the structure is more coherent, and the mathematical arguments are now more clearly justified.

 The changes made by the authors have strengthened the overall quality of the manuscript.

 In its current form, the article meets the standards for indexing.

Is the work clearly and accurately presented and does it cite the current literature?

Yes

If applicable, is the statistical analysis and its interpretation appropriate?

Yes

Are all the source data underlying the results available to ensure full reproducibility?

Yes

Is the study design appropriate and is the work technically sound?

Yes

Are the conclusions drawn adequately supported by the results?

Yes

Are sufficient details of methods and analysis provided to allow replication by others?

Yes

Reviewer Expertise:

analytic and univalent functions

I confirm that I have read this submission and believe that I have an appropriate level of expertise to confirm that it is of an acceptable scientific standard.

# Reviewer report: Reviewer response for version 1

**DOI:** 10.5256/f1000research.191117.r468727

**Published:** 2026-03-25

**Authors:** suha jumaa hammad

**Affiliations:** 1 University of Tikrit, Tikrit, Iraq

**Recommendation**: Accept the application with minor revision

**Manuscript title**: Bounds on Hankel Determinants with Fekete–Szegő Parameter for Bazilević Functions

 The author is to be highly commended for deriving explicit and well-structured bounds for the first four coefficients a2,a3,a4 and a5  which constitutes a significant and nontrivial contribution within the framework of Geometric Function Theory. Obtaining such higher-order coefficient estimates is inherently challenging, particularly for generalized subclasses defined via subordination. 

 Moreover, the investigation of the Fekete–Szegő inequality with a general parameter τ\tauτ reflects a deep understanding of the structural aspects of coefficient problems. The inclusion of this parameterized functional not only broadens the scope of the results but also enhances their applicability within the theory of univalent functions.

 In addition, the treatment of Hankel determinants represents a particularly valuable component of the paper. The derivation of bounds for second- and third-order Hankel determinants requires careful and delicate analysis due to the intricate dependence on coefficient interactions. The results obtained in this regard demonstrate substantial analytical effort and contribute meaningfully to the advancement of the field, especially in the context of generalized subclasses associated with Ma–Minda functions.

 All the arithmetic and analytical computations presented in the manuscript, including the derivation of the bounds for the coefficients a2,a3,a4,a5 and the estimation of the Hankel determinants, are mathematically correct.

 The conclusions are generally clear and summarize the main findings well.

**Minor corrections needed**.

 1-Although the manuscript is generally clear, some sentences are long and could be shortened for easier reading.

 2-The two special cases listed in Remark 1 (points 1 and 2) are essentially the same. To avoid redundancy and improve clarity, it is recommended to merge them into a single example. This will make the remark more concise and easier for readers to follow.

 3-References/Bibliography: The bibliography is valuable; however, it would be helpful to include a few more recent and relevant works to further strengthen the manuscript.

Is the work clearly and accurately presented and does it cite the current literature?

Yes

If applicable, is the statistical analysis and its interpretation appropriate?

Yes

Are all the source data underlying the results available to ensure full reproducibility?

Yes

Is the study design appropriate and is the work technically sound?

Yes

Are the conclusions drawn adequately supported by the results?

Yes

Are sufficient details of methods and analysis provided to allow replication by others?

Yes

Reviewer Expertise:

analytic and univalent functions

I confirm that I have read this submission and believe that I have an appropriate level of expertise to confirm that it is of an acceptable scientific standard, however I have significant reservations, as outlined above.

# Response

**Published:** 2026-03-27

**Authors:** Nathir Khaled

**Affiliations:** Mathematics, University of Anbar, Ramadi, Al Anbar Governorate, Iraq

**D**ear Prof...

 Best greetings.....

 I hope this message finds you well.

 Subject \ comments responses about the manuscript- F1000Res173313

 (Bounds on Hankel Determinants with Fekete-Szegö Parameter for Bazilević Functions)

 We sincerely thank for their valuable comments and constructive suggestions, which have helped us significantly improve the quality of our manuscript. We have carefully addressed all the points raised. Below, we provide a detailed point-by-point response.

 1-Although the manuscript is generally clear, some sentences are long and could be shortened for easier reading.

**Response 1:** We have shortened lengthy sentences throughout the manuscript to improve readability.

 2-The two special cases listed in Remark 1 (points 1 and 2) are essentially the same. To avoid redundancy and improve clarity, it is recommended to merge them into a single example. This will make the remark more concise and easier for readers to follow.

**Response 2:** We have merged these cases into one clear example.

 3-References/Bibliography: The bibliography is valuable; however, it would be helpful to include a few more recent and relevant works to further strengthen the manuscript.

**Response 3: **We have added several recent references (e.g., from 2024–2025) to strengthen the bibliography.

 Acknowledgments: Thankful to reviewers for their valuable corrections and important suggestions

# Reviewer report: Reviewer response for version 1

**DOI:** 10.5256/f1000research.191117.r468724

**Published:** 2026-03-20

**Authors:** Alina Alb Lupas

**Affiliations:** 1 University of Oradea, Oradea, Romania

**REVIEWER’s REPORT**

*On the paper*

**Bounds on Hankel Determinants with Fekete-Szegö Parameter for Bazilević Functions**

 by Nathir Khaled , Abdul Rahman S.Juma

 In this paper the authors establish the properties of a new subclass of functions that generalizes Yamaguchi and starlike functions: tight upper estimates on the initial coefficients, the Fekete-Szegö functional for parameters, and the second and third-order Hankel determinants involving a parameter τ >0.

 The results are new, correct and detailed. The paper is original and doesn’t contradict to ethical or policy issues, the question posed by authors is new and well defined, the methods used by authors are appropriate and well described, the data are sound and well controlled, the discussion and conclusions are well balanced, the title and abstract convey the obtained results, the writing is acceptable, the paper contains good scientific results.

 The paper doesn’t require a revision.

             Taking the above into consideration, I recommend the paper for indexing.

 18.03.2026                                                               

Is the work clearly and accurately presented and does it cite the current literature?

Yes

If applicable, is the statistical analysis and its interpretation appropriate?

Not applicable

Are all the source data underlying the results available to ensure full reproducibility?

Yes

Is the study design appropriate and is the work technically sound?

Yes

Are the conclusions drawn adequately supported by the results?

Yes

Are sufficient details of methods and analysis provided to allow replication by others?

Yes

Reviewer Expertise:

Complex Analysis

I confirm that I have read this submission and believe that I have an appropriate level of expertise to confirm that it is of an acceptable scientific standard.

# Response

**Published:** 2026-03-27

**Authors:** Nathir Khaled

**Affiliations:** Mathematics, University of Anbar, Ramadi, Al Anbar Governorate, Iraq

**Dear Prof...**

**Best greetings.....**

**I hope this message finds you well.**

**Subject \ comments responses about the manuscript-** **F1000Res173313**

**(Bounds on Hankel Determinants with Fekete-Szegö Parameter for Bazilević Functions)**

 We sincerely thank for their valuable comments and constructive suggestions, which have helped us significantly improve the quality of our manuscript. We have carefully addressed all the points raised. 

**Acknowledgments:** **Thankful to reviewers for their valuable corrections and important suggestions**

# Reviewer report: Reviewer response for version 1

**DOI:** 10.5256/f1000research.191117.r459194

**Published:** 2026-03-06

**Authors:** Afis Saliu

**Affiliations:** 1 University of the Gambia, Serrekuna, The Gambia

The authors introduce a Bazilevič-type family associated with the general Ma–Minda function and investigate several coefficient-related results. While the topic is potentially interesting, the manuscript requires substantial revision in terms of structure, clarity, and scholarly presentation before it can be considered for indexing.

 Below are my detailed observations and recommendations:

 \begin{itemize}

     \item[(1)] The abstract needs to be rewritten to make it concise, precise, and clearly aligned with the main objectives and contributions of the paper. At present, it does not adequately reflect the core findings.

     \item[(2)] The first paragraph of the introduction does not relate to the main results obtained in the paper.

     \item[(3)] Consequently, this paragraph should either be removed or substantially revised. Moreover, it is unclear how the class $\mathcal{S}$ admits representation (1); this point requires clarification and proper justification.

     \item[(4)] Overall, the introduction is not well written. There is no clear connection between the present work and existing literature, and the motivation for the study is insufficiently articulated. Parts of Section 1.1 should be systematically merged into the introduction to improve logical flow. In addition, grammatical errors in these sections must be carefully corrected.

     \item[(5)] There is a misuse of notation for Carathéodory functions in equations (5) and (6). The notation should be made consistent and mathematically accurate.

     \item[(6)] The manuscript lacks sufficient and relevant references to support the stated results and contextualize the contribution within the existing body of work.

     \item[(7)] The properties of Ma–Minda functions listed on page 4 are incomplete. These properties should be presented in a structured and itemized form to improve clarity and readability.

     \item[(8)] Throughout most parts of the manuscript, the authors assume $f\in\mathcal{S}$ instead of $f\in A$. The choice between $f\in A$ and $f\in\mathcal{S}$ significantly affects the definitions and subsequent results. For example, in the definition of $\mathcal{ST}$, the assumption $f\in\mathcal{S}$ implicitly suggests that every univalent function is starlike, which is not true in general. For instance, the function

     $f(z)=z+\frac{1}{2}z^{2}$

     is univalent but not starlike with respect to the origin in the open unit disk.

     \item[(9)] There are numerous punctuation errors throughout the manuscript. The paper requires careful proofreading.

     \item[(10)] The overall organization of the paper is weak, particularly in the introduction and Section 1.1. A clearer structural framework is necessary.

     \item[(11)] In the inclusion relation following equation (11), the symbols $\mathcal{B}_{1}(\delta)$ and $\mathcal{B}(\delta)$ are used without definition.

     \item[(12)] In Lemmas 3.1--3.4, the symbol $\mathcal{CR}$ appears without prior definition.

     \item[(13)] Lemma 3.1 is not properly typeset and should be reformatted for clarity and correctness.

     \item[(14)] The short statement preceding the main results appears disconnected from the surrounding discussion. It should either be integrated properly or removed.

     \item[(15)] In Remark 1, Special Cases 1 and 2 are identical. This redundancy should be corrected.

     \item[(16)] The origin of the assumption $\tau>0$ in the final paragraph before Section 3.2 is unclear and requires justification.

     \item[(17)] The authors should explicitly state whether their results are sharp. If so, appropriate justification and extremal functions must be provided; otherwise, the validity and optimality of the coefficient bounds remain uncertain.

     \item[(18)] Finally, the manuscript contains numerous grammatical errors that must be thoroughly corrected to meet academic standards.

 \end{itemize}

 \begin{center}

 \section*{Recommendation}

 \end{center}

 In its current form, the manuscript requires major revision, particularly with respect to mathematical rigor, organization, clarity of definitions, and linguistic accuracy. A careful restructuring and substantial editorial improvement are strongly recommended.

Is the work clearly and accurately presented and does it cite the current literature?

No

If applicable, is the statistical analysis and its interpretation appropriate?

Not applicable

Are all the source data underlying the results available to ensure full reproducibility?

No source data required

Is the study design appropriate and is the work technically sound?

No

Are the conclusions drawn adequately supported by the results?

Partly

Are sufficient details of methods and analysis provided to allow replication by others?

Yes

Reviewer Expertise:

Complex Analysis (Geometric function theory)

I confirm that I have read this submission and believe that I have an appropriate level of expertise to state that I do not consider it to be of an acceptable scientific standard, for reasons outlined above.

# Response

**Published:** 2026-03-27

**Authors:** Nathir Khaled

**Affiliations:** Mathematics, University of Anbar, Ramadi, Al Anbar Governorate, Iraq

**Dear Prof...**

**Best greetings.....**

**I hope this message finds you well.**

**Subject \ comments responses about the manuscript-** **F1000Res173313**

**(Bounds on Hankel Determinants with Fekete-Szegö Parameter for Bazilević Functions)**

 We sincerely thank for their valuable comments and constructive suggestions, which have helped us significantly improve the quality of our manuscript. We have carefully addressed all the points raised. Below, we provide a detailed point-by-point response.

**Response 1:** We have rewritten the abstract to be more concise and focused on the main objectives, methodology, and key results. The revised abstract now clearly highlights the class definition, the derived sharp bounds, and the significance of the results.

**Response 2 & 3:** We have removed the irrelevant opening paragraph and clarified the representation of class S. Equation (1) now explicitly states the normalization conditions f(0)=0 and f′(0)=1, and we have added a brief justification for the series form.

**Response 4: **We have restructured the introduction entirely. Section 1.1 has been merged into the introduction to create a logical flow. We added a clear motivation, linked our work to existing literature, and corrected grammatical errors throughout.

**Response 5:** We have corrected the notation. The Carathéodory function is now consistently denoted by p(z) instead of  CR, and equations (5) and (6) have been revised accordingly.

**Response 6: **We have added several recent and relevant references to better contextualize our work within the existing literature, particularly regarding Hankel determinants, Fekete–Szegő functionals, and Ma–Minda functions..

**Response 7:** We have restructured the properties of the Ma–Minda function into a clear, itemized list for improved readability.

**Response 8:** We have corrected this issue throughout the manuscript. The definitions now clearly use f ∈ A where appropriate, and the class S is reserved for univalent functions. The definition of starlike functions has been corrected to require f ∈ S only after the geometric condition is imposed.

**Response 9:** The entire manuscript has been carefully proofread, and punctuation errors have been corrected.

**Response 10:** We have reorganized the manuscript. The introduction now integrates all preliminary concepts, and the structure follows a logical progression: introduction → lemmas → main results (definition, coefficient bounds, Fekete–Szegő, Hankel determinants) → conclusion.

**Response 11:** We have added clear definitions for these symbols following equation (11).

**Response 12 & 13: **We have replaced CR with the standard notation P (Carathéodory class) throughout the lemmas. Lemma 1 has been reformatted for clarity.

**Response 14:** This statement has been integrated into the introduction or removed as appropriate.

**Response 15:** We have merged these two cases into a single example and corrected the remark accordingly.

**Response 16**: We have added a justification for τ > 0 in Section 3.4, explaining that it serves as a generalized Fekete–Szegő parameter and that the case τ = 1 reduces to the classical Hankel determinant.

**Response 17: **We have revised the manuscript to explicitly state sharpness where applicable and provided extremal functions (e.g., the Koebe function and Möbius transformations) for the coefficient bounds.

**Response 18:** A thorough language revision has been performed to meet academic standards.

**Acknowledgments:** **Thankful to reviewers for their valuable corrections and important suggestions**
